# Direct electrodeposition of lithium titanate as a lithium-ion battery anode active material in propylene carbonate solution containing titanyl compounds

**DOI:** 10.1039/d5ra06413a

**Published:** 2025-09-25

**Authors:** Fatma Çambay Kuban, Kadir Pekmez

**Affiliations:** a Graduate School of Science and Engineering, Nanotechnology and Nanomedicine Division, Hacettepe University Ankara Turkey; b Turkish Aerospace 06980 Ankara Turkey; c Department of Chemistry, Hacettepe University Ankara Turkey pekmez@hacettepe.edu.tr

## Abstract

Lithium titanium oxide (LTO) type materials for lithium-ion (Li-ion) batteries have become an alternative to the typically used graphitic-based materials as anode materials due to their better safety performance and longer life cycles. In the literature, LTO structures such as Li_4_Ti_5_O_12_, Li_2_Ti_3_O_7_ and Li_2_Ti_3_O_7_/Li_2_TiO_3_ composites with different Li/Ti ratios have been synthesized by solution and solid phase methods such as sol–gel, spray drying, spray pyrolysis and, ultrasonic spray pyrolysis at elevated temperatures using TiO_2_ and Li_2_CO_3_ as starting materials, and their electrochemical performances have been tested. This study demonstrated that electrochemical deposition can directly deposit LTO anode electrode materials using TiOSO_4_ and TiO(ClO_4_)_2_ precursor compounds in Propylene Carbonate (PC) solvent containing LiClO_4_ supporting electrolyte at room temperature. TiO(OH)_2_ and TiOOH, which are formed by the reactions of the unstable TiO^+^ ion formed by electrochemical reduction with TiO^2+^ in solution and adsorbed on the electrode surface and the OH^−^ ion formed by the electroreduction of water, and TiO(OH)_2_ and TiOOH precipitated on the electrode surface, interact with the excessive amount of Li^+^ ions in solution to form lithium titanate. Since electrochemical reduction occurs between 2.4 V and 1.2 V, lithium can be incorporated during electrodeposition, resulting in the formation of various lithium titanate phases (Li_4_Ti_5_O_12_, Li_2_Ti_3_O_7_ and Li_2_Ti_3_O_7_/Li_2_TiO_3_ composite). Electrodeposited Lithium Titanate (ED-LTO) obtained in this way has been characterized using cyclic voltammetry, chronopotentiometry, EIS, XRD, XPS, Raman spectroscopy, and FESEM-EDX techniques. A full cell was fabricated using ED-LTO/LiFePO_4_.

## Abstract 2

Lithium titanium oxide (LTO) anode materials for lithium-ion (Li-ion) batteries have become an alternative to the typically used graphitic-based materials as anode materials due to their better safety performance and longer life cycles.

## Graphical abstract

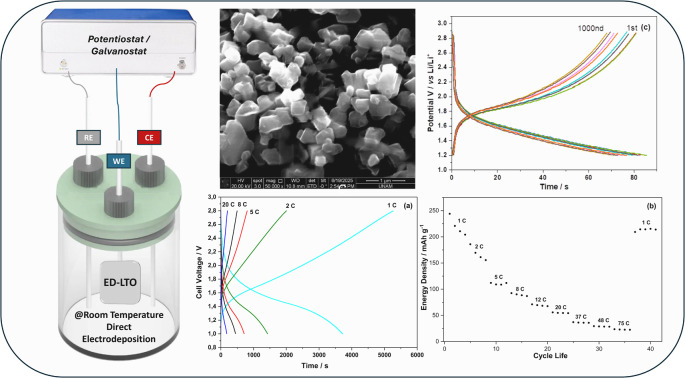

## Introduction

Due to the excessive consumption of fossil fuels and the associated environmental pollution and global warming, the trend toward renewable sources such as solar and wind energy has rapidly increased in the last 20 years.^1–4^ These intermittent alternative energy sources require large-scale energy storage devices to store them as electrical energy. Among the various technologies available for energy storage, lithium-ion (Li-ion) batteries stand out for their superior energy density. While lithium-ion batteries have been used since the 1990s for portable electronic devices such as mobile phones, digital cameras and laptops, they are becoming widespread for large-scale energy storage in electric vehicles (EVs) in the transportation sector and smart grids in the renewable energy field. Now, graphitic carbon in different forms has been widely used as anode material for lithium-ion (Li-ion) batteries.^5–8^ However, the formation of a solid–electrolyte interface layer (SEI) on carbon anodes at voltages below 0.8 V leads to impedance increases, rechargeable capacity decreases and consequently shorter life cycles in these batteries. Therefore, lithium titanium oxide (LTO) type materials have been candidates as anode materials for lithium-ion (Li-ion) batteries due to their better safety performance and long life cycles.^5^ One such LTO material, spinel type Li_4_Ti_5_O_12_, has been investigated as an alternative anode material and found to have a wide plateau at 1.5 V and an excellent charge–discharge cycle due to its structural stability during the Li-ion insertion process.^5,9,10^ However, LTO's low electronic conductivity due to its insulating ionic crystal structure is one of its major drawbacks as an anode material.^5,11^ To improve its electrical conductivity, carbon-based materials are often added to LTO, which can improve its electrochemical properties, such as charge–discharge characteristics.^5,11–14^

Lithium titanium oxides (LTO) such as Li_4_Ti_5_O_12_, Li_2_Ti_3_O_7_ and Li_2_Ti_3_O_7_/Li_2_TiO_3_ composites with different Li/Ti ratios have been synthesized by solution and solid phase methods such as sol–gel, spray drying, spray pyrolysis and, ultrasonic spray pyrolysis at high temperatures (600–1100 °C) using TiO_2_, Li_2_CO_3_ as the starting materials and finally, their electrochemical properties have been investigated for their use as battery anode materials.^5,15–19^ On the other hand, a different type of lithium titanate, ramsdellite type Li_2_Ti_3_O_7_ (RLTO), already has good electrical conductivity itself and can therefore, be used as an anode without the need for carbon doping.^5–10^ Moreover, the electrochemical properties of RLTO anodes prepared using solid-state reaction can be highly improved.^5,11–13^ It has been shown that the ramsdellite series Li_1+*x*_Ti_2−2*x*_O_4_ exists between the LiTi_2_O_4_ and Li_2_Ti_3_O_7_ compounds at high temperatures, and when cooled, these two phases can be maintained up to room temperature.^5,10^ A cyclic voltammogram of the spinel Li_4_Ti_5_O_12_ was reported to show a large reversible peak at about 1.55 V (relative to Li/Li^+^). Ramsdellite phases showed a similar reversible peak at a lower than 1.5 V. However, a series of additional reversible peaks up to 2.0 V were reported to be observed. As the value of *x* in Li_1+*x*_Ti_2−2*x*_O_4_ increases, these extra peaks shift to lower voltages. For Li_2_Ti_3_O_7_, during charge and discharge, the potential varies in the range of 1.5 to 2.5 V and high capacities, typically approaching 200 mA h g^−1^, have been achieved.^5,10^ Among the alternative anode candidates to graphite, spinel lithium titanate oxide (Li_4_Ti_5_O_12_, LTO) has been considered the most promising due to its excellent safety properties and high cycle life. Unlike the graphite anode, which expands up to 10% volume during charging, Li_4_Ti_5_O_12_ allows the entry of three lithium ions per formula unit, thus achieving a theoretical capacity of 175 mA h g^−1^, while negligible volume change occurs during charge and discharge.^5,10,20–23^ During charging, the three Ti^4+^ ions in the spinel structure are reduced to Ti^3+^ ions; three Li^+^ are introduced into the structure to compensate for the lack of positive charge deficiency, thus transforming the structure from spinel-LTO to rock salt Li_7_Ti_5_O_12_.^3–7,12,15^

1 Li_4_Ti_5_O_12_ + 3Li^+^ + 3e ↔ Li_7_Ti_5_O_12_

This equation shows that a typical reversible electrochemical reaction occurs between spinel-LTO and rock salt-LTO during lithium insertion and extraction processes.

Among traditional LTO synthesis methods, techniques such as sol–gel and hydrothermal, which are typically performed at relatively low temperatures, are also widely used. The sol–gel method generally requires pre-calcination at 300–600 °C followed by final calcination at 600–800 °C, while hydrothermal synthesis typically involves long-duration reactions at 120–200 °C and an additional calcination step. These methods can be limited by high energy consumption and lengthy processing times. In contrast, the direct electrodeposition method presented in this study enables the preparation of LTO films in a single step at room temperature, without requiring any thermal treatment. As a result, both energy consumption and total processing time are significantly reduced. Furthermore, the ability to directly control phase composition and morphology during electrodeposition enhances the efficiency and scalability of the method. In this study, it has been shown that LTO anode electrode materials can be deposited at room temperature using TiOSO_4_ and TiO(ClO_4_)_2_ precursor compounds by direct electrochemical constant potential and constant current electrolysis methods in propylene carbonate (PC) solvent containing LiClO_4_ supporting electrolyte. The electrodeposited lithium titanate (ED-LTO) anode material obtained in this way has been characterized by electrochemical methods such as cyclic voltammetry, chronopotentiometry, EIS, XRD, XPS, Raman spectroscopy, and FESEM-EDX techniques.

## Experimental method

In electrochemical experiments, propylene carbonate (PC) (Merck) was used as a solvent, and LiClO_4_ (Fluka) was used as a supporting electrolyte. TiOSO_4_ (Sigma-Aldrich) was used as a titanium source and since it was not sufficiently soluble in PC solvent, it was converted to TiO(ClO_4_)_2_ by metathesis reaction in PC solvent using Ba(ClO_4_)_2_ (Merck Emsure). The solid BaSO_4_ formed during this reaction was filtered off, and the resulting solutions of TiO(ClO_4_)_2_ in a PC with a known concentration were prepared, these solutions were used in all electrochemical experiments. Commercial LTO (spinel Li_4_Ti_5_O_12_) and LiFePO_4_ were purchased from Nanografen. Cyclic voltammetry (CV), electrochemical deposition (ED), charge–discharge tests, and impedance spectroscopy (EIS) experiments were carried out using CHI660 (CH Instruments) in a three-electrode electrochemical cell consisting of a working electrode, reference electrode and counter electrode. Pt (Aldrich), Ti (Goodfellow) and graphite (SGL Carbon) disc or sheet electrodes were used as working electrodes in different studies. An Ag/AgCl electrode containing saturated AgCl and LiClO_4_ in PC was placed in a glass compartment separated by a glass fritted disc and used as a reference electrode. The voltage of the Ag/AgCl reference electrode in the PC was measured to be 3.2 V *versus* Li/Li^+^ pair and the voltages in the electrochemical experiments were given with respect to the Li/Li^+^ reference as it is convenient for practical purposes. The coated electrodes were analyzed using XRD (PANalytical), FESEM-SE (FEI-NANOSEM and PANalytical X'Pert PRO), RAMAN (BWTEK Fiber Optic Raman System), XPS (Specs-Flex). Depth profile analysis was performed using XPS. Commercial LTO, carbon black and PVDF were mixed in an 80 : 10 : 10 weight ratio to prepare a slurry, respectively, which was drop-cast onto carbon paper (GDL39) and dried. The resulting electrode was further dried at 60 °C for 24 h to remove residual solvent. For the full-cell assembly, commercial LiFePO_4_ was coated onto carbon paper with carbon black and PVDF in the same 80 : 10 : 10 ratio, respectively. The LiFePO_4_/ED-LTO cell was then assembled and cycled between 1.0 and 2.8 V.

## Results and discussion

### Electrochemical deposition and characterization of LTO

**Fig. 1:**
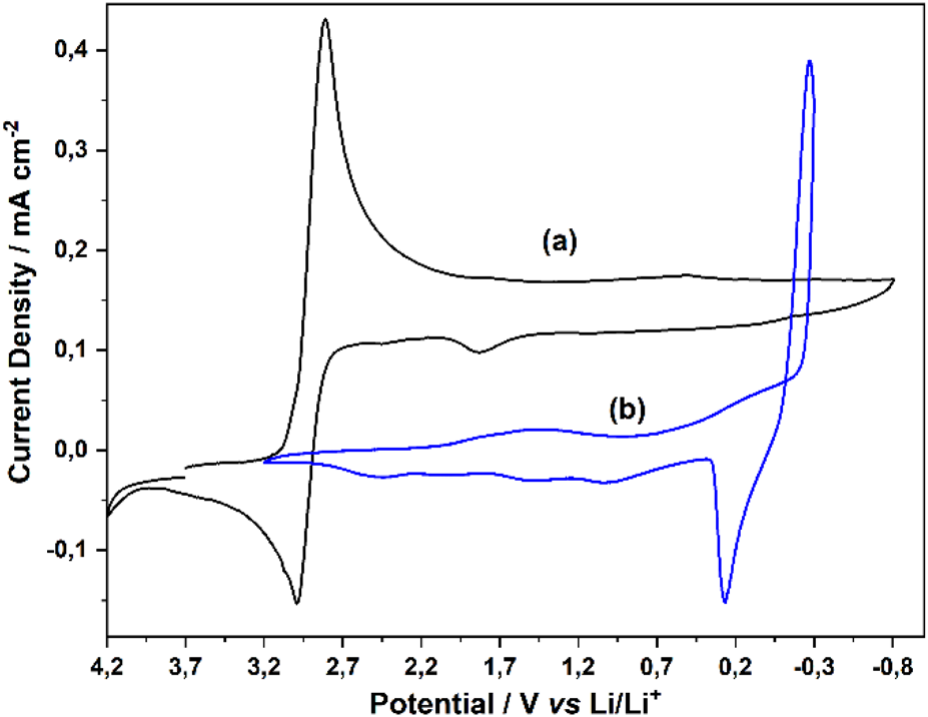
(a) CV obtained on Pt disc electrode in PC solution containing 0.100 M TiO(ClO_4_)_2_, (b) 0.100 M LiClO_4_, scan rate 100 mV s^−1^.

To obtain lithium titanium oxide (LTO) anode electrode materials from TiO(ClO_4_)_2_ precursor compounds using direct electrochemical methods, in the first step, the cyclic voltametric (CV) behavior of the precursor compounds was studied in propylene carbonate (PC) solvent containing LiClO_4_ supporting electrolyte. Fig. 1(a) shows the CV obtained with Pt disc electrode in PC solution containing 0.100 M TiO(ClO_4_)_2_. As seen in the CV, the titanyl TiO^2+^ (Ti^4+^) ion in the solution is reduced to TiO^+^ (Ti^3+^) at 2.9 V in the negative direction starting from 3.5 V with respect to the Li/Li^+^ reference electrode. Since only TiO(ClO_4_)_2_ is present in this solution, the peak belonging to the reduction of TiO^2+^ ion, which is the main electrochemical process, is observed. However, the wide band observed at the residual current level between +1.7 V and +0.2 V belongs to the reduction of TiO^+^ (Ti^3+^) to metallic Ti. This weak transformation has also been observed in the literature in acidic solutions containing TiCl_4_ in PC.^24^

Fig. 1(b) shows the CV obtained with Pt disc electrode in PC solution containing 0.100 M LiClO_4_. This solution contains only LiClO_4_, which is used as a supporting electrolyte and Li^+^ source in all electrochemical experiments performed hereafter. The CV in Fig. 1(b) shows the conversion of Li^+^ to metallic lithium by reduction on the Pt electrode surface at around 0.0 V with respect to the Li/Li^+^ reference electrode and the stripping of this metallic Li by oxidation in the reverse cycle.

According to eqn (2), the titanyl TiO^2+^ (Ti^4+^) ion dissolved in PC is reduced to TiO^+^ (Ti^3+^) at 2.9 V on the Pt electrode surface.

2 TiO^2+^ + e^−^ → TiO^+^

**Fig. 2:**
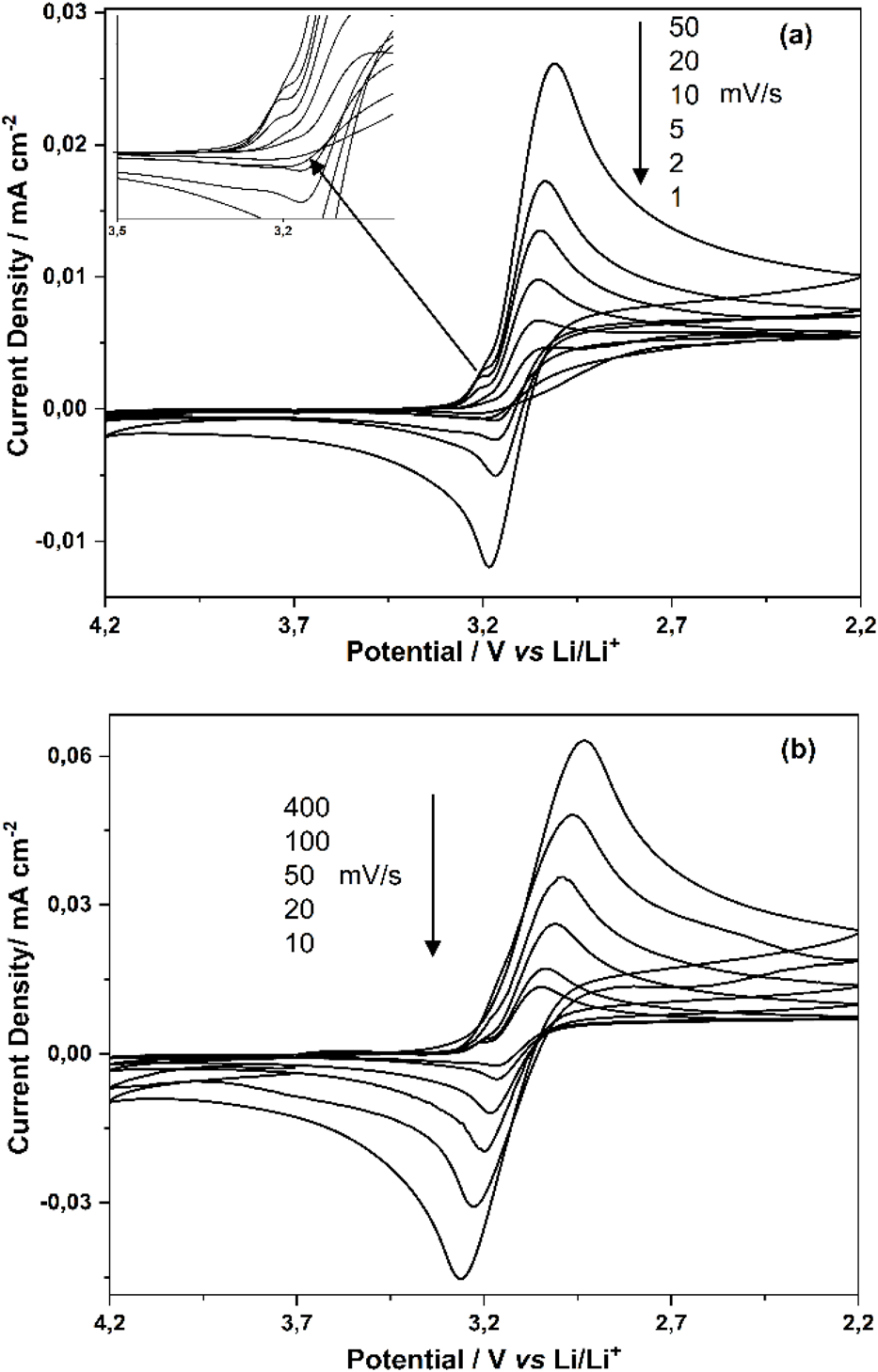
CVs obtained at different scan rates (a) 1, 2, 5, 10, 20, 50 mV s^−1^ (b) 10, 20, 50, 100, 400 mV s^−1^ on Pt disc electrode in PC solution containing 0.100 M TiO(ClO_4_)_2_.

In order to understand this electrochemical reduction process, CVs were taken at different potential scan rates (*v*) and narrower voltage range (between 4.2 V and 2.2 V) and these measurements are shown in Fig. 2(a) (1, 2, 5, 10, 20, 50 mV s^−1^) and Fig. 2(b) (10, 20, 50, 100, 200, 400 mV s^−1^). Above scan rates of 20 mV s^−1^, the difference between the reduction peak potential of TiO^2+^ ion and the oxidation potential of TiO^+^ in the reverse scan (Δ*E*: *E*_pa_ − *E*_pk_) gradually increases as the scan rate increases. On the other hand, when the scan rates are decreased from 400 mV s^−1^ to lower scan rates (50 mV s^−1^, 10 mV s^−1^ and below 1 mV s^−1^), the ratio of the oxidation current of TiO^+^ in the reverse cycle and the reduction peak currents of TiO^2+^ ion (*I*_pa_/*I*_pc_) decreases gradually and almost no anodic peak current is observed at scan rates of 5, 2, 1 mV s^−1^.

According to these electrochemical data, the electrochemical reduction reaction of TiO^2+^ + e^−^ → TiO^+^ on the Pt electrode surface and in PC solvent shows a quasi-reversible behavior; the reduction product TiO^+^ is unstable and easily consumed by successive chemical reactions with other species present in the solvent and solution. In addition, the CV in Fig. 2(a) (see inside graphics) shows a pre-peak at the first appearance of the reduction current around 3.2 V (at low scan rates of 5, 10, 20 mV s^−1^) during the reduction of TiO^2+^ to TiO^+^. This shoulder-shaped pre-peak indicates that the TiO^+^ ion formed by electroreduction is strongly adsorbed on the Pt electrode surface.^25^

**Fig. 3:**
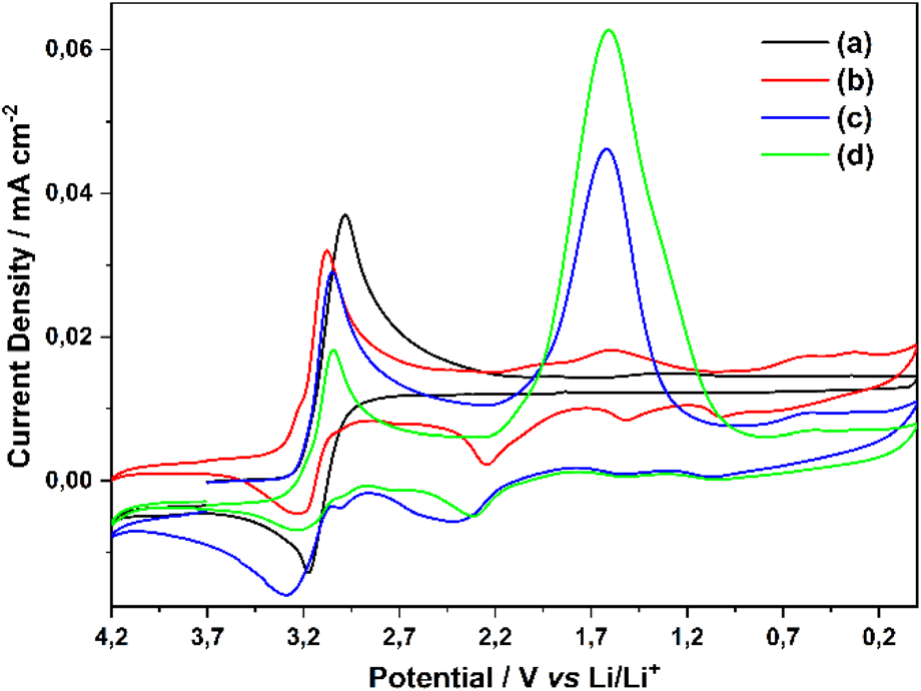
(a) CV obtained on Pt disc electrode in 0.100 M TiO(ClO_4_)_2_ PC solution, (b) CV obtained after addition of 0.1 M H_2_O and LiClO_4_ to the solution in (a), (c) CVs of solutions obtained by adding 0.5 M LiClO_4_ (d) 1.0 M LiClO_4_ to the solution in (b). respectively, scan rate 100 mV s^−1^.

When 0.1 M H_2_O and 0.1 M LiClO_4_ are added to the PC solution containing 0.100 M TiO(ClO_4_)_2_, the CV of the resulting solution taken with a Pt disc electrode shows the TiO^2+^/TiO^+^ reduction peak at 2.9 V, as well as new reduction peaks at 1.7 V and their corresponding oxidation peaks in the reverse cycle (Fig. 3(a) and (b)). When LiClO_4_ is continued to be added, it is observed that the current of the new reduction peak at 1.7 V increases with the increase in LiClO_4_ concentration and the oxidation peaks of the products at 2.2 V in the reverse scan become increasingly prominent. However, the back-oxidation peak of the main product TiO^+^ at 3.2 V decreases with the addition of LiClO_4_.

When a PC solution containing TiO(ClO_4_)_2_ is added to a relatively low concentration of water and LiClO_4_ as a supporting electrolyte (which also acts as a source of lithium), TiO^2+^ + e^−^ → TiO^+^. Besides the TiO^+^ reduction reaction, many sequential electrochemical and chemical reactions occur, and the electrochemical behavior changes completely. The following equations can give possible reactions between approximately 2.4 V and 1.2 V.

2 TiO^2+^ + e^−^ → TiO^+^

3 2H_2_O + 2e^−^ → 2OH^−^ + H_2_

4 TiO^2+^ + 2OH^−^ → TiO(OH)_2_

5 TiO^+^ + OH^−^ → TiOOH

TiO(OH)_2_ and TiOOH, which are formed by the reactions of the unstable TiO^+^ ion formed by electrochemical reduction with TiO^2+^ in solution and adsorbed on the electrode surface and the OH^−^ ion formed by the electroreduction of low concentrations of water and precipitated and deposited on the electrode surface, interact with the excessive amount of Li^+^ ions in solution to form lithium titanate. Since electrochemical reduction between voltages of 2.4 V and 1.2 V also takes place, lithium can be inserted by electroreduction during electrochemical deposition (electroprecipitation) and different types of lithium titanate LTO (Li_4_Ti_5_O_12_, Li_2_Ti_3_O_7_ and Li_2_Ti_3_O_7_/Li_2_TiO_3_ composite)^5,9,10,13,17,18,26^ can be formed together. This mechanism, which we proposed based on the cyclic voltametric data, is in agreement with the XRD, XPS, Raman spectroscopy, SEM EDX characterization results given in the next section.

Furthermore, if LiOH is added to a PC solution (Fig. S1(a) and (b)) containing 0.100 M TiO(ClO_4_)_2_, 0.100 M water and 0.500 M LiClO_4_, the solution is made basic and when the CV of this solution is taken, the peak at 2.9 V for the reduction of TiO^2+^ ion completely disappears and only the broad peak between 2.2 V and 1.2 V for the electrochemical LTO formation is observed (Fig. S1(c)). This result supports the formation of TiO(OH)_2_, TiOOH proposed in eqn (4) and (5).

During the cyclic voltametric experiment in a PC solution containing 0.100 M TiO(ClO_4_)_2_, 0.100 M water and 0.500 M LiClO_4_, the voltage applied to the Pt disk electrode was stopped and held at 0.7 V beyond the electro-reduction peak and electrolysis was performed for certain periods to obtain electrochemically deposited LTO. After the end of electrolysis, the voltage scan was continued at the same voltage, and it was observed that the current of the oxidation peak indicating Li^+^ discharged from the LTO structure increased in proportion to the increase in the electrolysis time (Fig. S2(a)–(c)). This study used Pt, Ti, graphite disc and sheet electrodes in PC solution containing 0.100 M TiO(ClO_4_)_2_, 0.100 M water and 0.500 M LiClO_4_ for electrochemical deposition of LTO. The electrochemically deposited lithium titanium oxide (ED-LTO) coated electrodes were characterized by electrochemical (CV, charge–discharge, EIS) and XRD, XPS, Raman, and SEM-EDX methods described hereafter. Electrochemical deposition processes were carried out by conventional constant voltage (potentiostatic) and voltage-limited constant current (galvanostatic) electrolysis. The voltage program applied to the working electrode in the voltage-limited galvanostatic method is similar to the voltage program of the chronopotentiometric charge–discharge test.

**Fig. 4:**
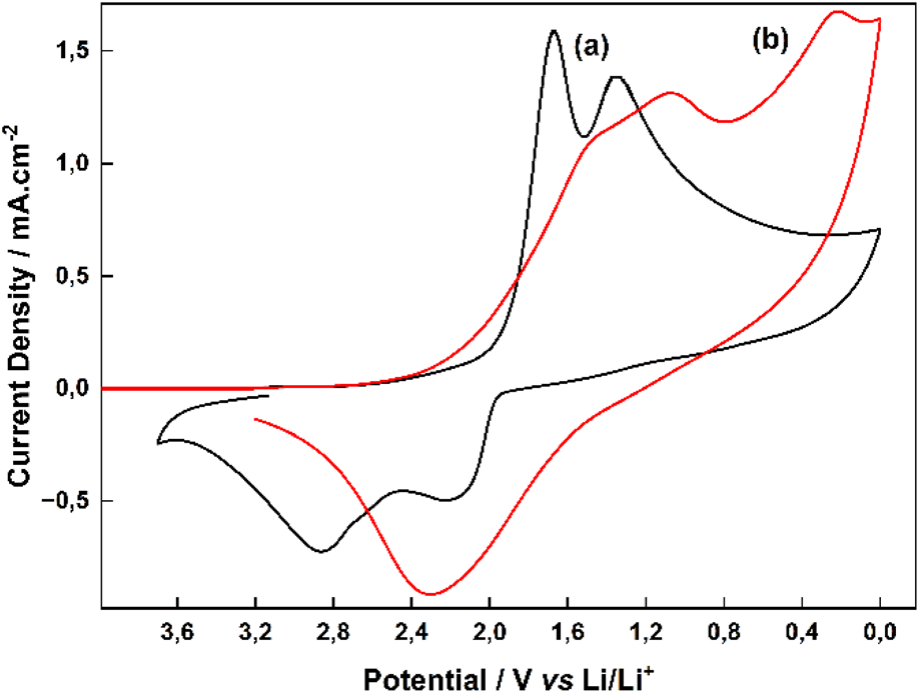
CVs of ED-LTO coating consisting of Li_2_Ti_3_O_7_ and Li_2_Ti_3_O_7_/Li_2_TiO_3_ composites deposited on (a) Pt (b) Ti disc electrode surface in 0.5 M LiClO_4_–PC blank solution, scan rate 10 mV s^−1^.

In order to investigate the electrochemical behavior of ED-LTO, in the first step, LTO was deposited on Pt and Ti disc electrode surfaces by constant voltage electrolysis at 0.7 V in PC solution containing TiO(ClO_4_)_2_, a low concentration of water and LiClO_4_ supporting electrolyte. Cyclic voltammograms of the ED-LTO coatings obtained in this way, washed and dried with acetonitrile and PC solvents and then taken in blank solution containing 0.5 M LiClO_4_ are shown in Fig. 4(a) and (b). During electrochemical deposition, lithium is introduced into the LTO structure by electroreduction process and different types of lithium titanate (Li_4_Ti_5_O_12_, Li_2_Ti_3_O_7_, and Li_2_Ti_3_O_7_/Li_2_TiO_3_ composite)^5,9,10,13,17,18^ are formed together. The electro-reduction reactions of these LTO species are generally given in the literature as follows.^19,27–29^Li_4_Ti_5_O_12_ + 3Li^+^ + 3e^−^ → Li_7_Ti_5_O_12_ (ref. **27** and **29**)Li_7_Ti_5_O_12_ + 2Li^+^ + 2e^−^ → Li_9_Ti_5_O_12_ (full reduction)^27^Li_2_Ti_3_O_7_ + *x*Li^+^ + *x*e^−^ → Li_2+*x*_Ti_3_O_7_ (*x* = 0.6 maximum)^28,29^Li_2_TiO_3_ (electrochemically inactive)^19^

Different types of LTO, including ramsdellite (RLTO), can undergo Li^+^ intercalation without any structural change. Electrochemical reduction reactions can introduce up to 3 mol Li^+^ per mole into the Li_4_Ti_5_O_12_ (spinel LTO) structure, while up to 0.6 mol Li^+^ per mole into the Li_2_Ti_3_O_7_ (RLTO) structure has been reported. Electrochemical intercalation reactions are more efficient than chemical intercalation, and as a result, the valence of the titanium ion in the structure decreases from +4 to +3.^27–29^

The reduction of ED-LTO species consisting of Li_4_Ti_5_O_12_, Li_2_Ti_3_O_7_, and Li_2_Ti_3_O_7_/Li_2_TiO_3_ composites deposited on the Pt disc electrode surface at CV in Fig. 4(a) is observed between 2.0 V and 1.2 V. During these electrochemical reduction processes, Li^+^ ion intercalation into the LTO structure leads to the transformation into Li_7_Ti_5_O_12_ and Li_2+*x*_Ti_3_O_7_ conducting species. While taking the cyclic voltammogram, when returning to the scan from 0.0 V, oxidation peaks attributed to the delithiation of Li^+^ ions are observed between 1.2 V and 3.2 V. The CV in Fig. 4(b) shows the electrochemical behavior of the same ED-LTO at the Ti disc electrode. On the Ti electrode surface, the peaks broaden as the two main reduction peaks shift to more negative voltages. A broad single peak is also observed for delithiation in the reverse scan. This indicates that the ED-LTO deposited on the Ti electrode surface has a slightly different composition and lower conductivity.

**Fig. 5:**
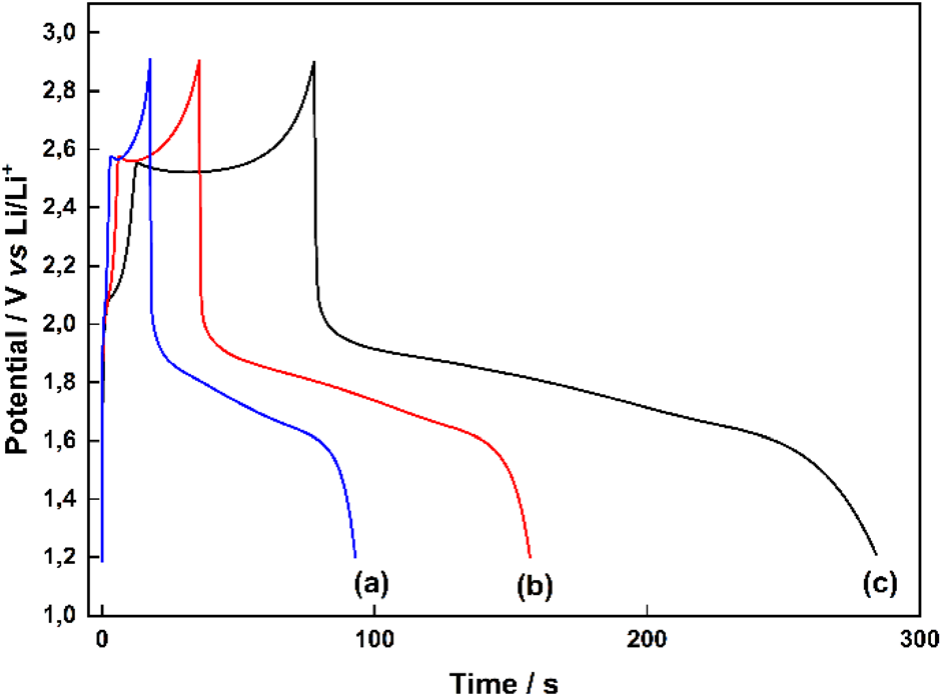
Chronopotentiogram of ED-LTO electrodeposited at 0.7 V for (a) 10 min, (b) 15 min (c) 25 min durations in PC solution containing 0.100 M TiO(ClO_4_)_2_, 0.1 M water and 0.5 M LiClO_4_ on the Pt disc electrode surface in the same solution at a current density of 0.02 mA cm^−2^ and in the range of 1.2 V to 2.9 V.

Chronopotentiometric curves of ED-LTO deposited by electrolysis at 0.7 V at different electrolysis times in PC solution containing 0.100 M TiO(ClO_4_)_2_, 0.1 M water and 0.5 M LiClO_4_ on the Pt disc electrode surface in the same solution are shown (Fig. 5). As the electrolysis time, the discharge times between 1.2 V and 2.9 V gradually increase with the amount of ED-LTO deposited, while the charging time takes longer than the discharge time due to the continuation of electrochemical deposition with the charging process between 1.2 V and 1.8 V in the charging cycle. In fact, in PC solution containing TiO(ClO_4_)_2_, water and LiClO_4_, LTO can be deposited electrochemically by galvanostatic charge–discharge method using a chronopotentiometric voltage program, and simultaneously Li^+^ intercalation and delithiation are provided to the LTO structure by electroreduction and different types of lithium titanate (Li_4_Ti_5_O_12_, Li_2_Ti_3_O_7_, and Li_2_Ti_3_O_7_/Li_2_TiO_3_ composite) are formed together.

**Fig. 6:**
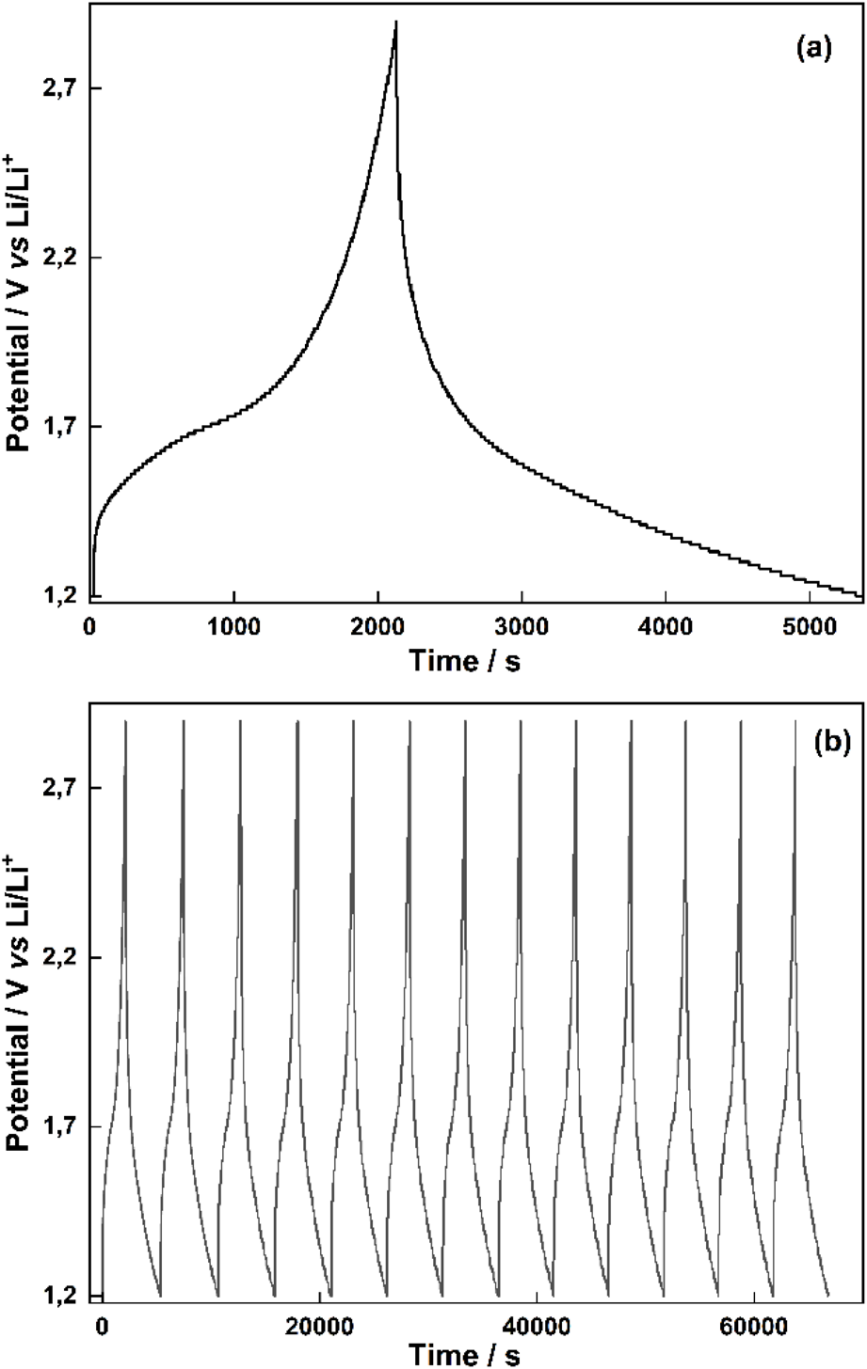
(a) Single cycle and (b) multi-cycle charge–discharge curves of ED-LTO electrodeposited at 0.7 V for 30 min in PC solution containing 0.100 M TiO(ClO_4_)_2_, 0.1 M water and 0.5 M LiClO_4_ on the Ti plate electrode surface at a current density of 0.02 mA cm^−2^ and in the range from 1.2 V to 2.9 V in 0.1 M LiClO_4_–PC blank solution.

The charge–discharge curves of ED-LTO electrodeposited at 0.7 V for 30 min in a PC solution containing 0.100 M TiO(ClO_4_)_2_, 0.1 M water and 0.5 M LiClO_4_ on the surface of Ti sheet electrode in 0.1 M LiClO_4_–PC blank solution are shown (Fig. 6). It is observed that this electrode exhibits the characteristic charge–discharge profile of LTO over a wide potential range of 1.2–2.9 V when the charge–discharge test was performed at a rate of about 2C. It maintains this profile over multiple cycles (Fig. 6(b)). When the capacity values were calculated, a 166 mA h g^−1^ value was obtained at 2C. The characteristic voltage profile of Li_4_Ti_5_O_12_, Li_2_Ti_3_O_7_, and Li_2_Ti_3_O_7_/Li_2_TiO_3_ composite (ED-LTO) during charge–discharge cycling at 2C is presented. The voltage curve exhibits a plateau of around 1.7 V during discharge; the charge profile peaks at around 2.9 V before gradually transitioning to the discharge phase, maintaining flatness even at this high C rate. The characteristic plateau region around 1.7 V during charge–discharge expands due to the composite structure of ED-LTO and the inclusion of different LTO species, which is typical for lithium titanate electrodes and reflects the two-phase reaction mechanism and excellent structural stability during lithium insertion/de-insertion. The symmetrical shape of the charge and discharge curves indicates good reversibility and low polarization at this high-rate condition, highlighting the suitability of lithium titanate for fast charging applications. This study demonstrated that ED-LTO deposited on the Ti electrode can be charged–discharged at high rates from 2C to 72C (Fig. S3). As the C-rate increases, the discharge time shortens, and voltage plateaus become less pronounced. When the rate capability of ED-LTO is analyzed against the energy density in increasing cycles at various C-rates, there is no significant loss of capacity as the C-rate increases, and no significant loss in capacity is observed when returning to 2C, indicating that ED-LTO exhibits structural stability and is reversible. In addition, as a result of the cycling test, 85% coulombic efficiency at 48C-rate and 95% cycle life at the end of 1000 cycles are obtained, indicating that the material works reversibly over long cycles and thus exhibits long cycle performance.

When ED-LTO on PV15 is examined by charge–discharge analysis, it is seen that it operates in a wide range of 2.1–230C (Fig. S4). Although the voltage profile becomes steeper as the C-rate increases, it exhibits a symmetrical profile. This indicates that ED-LTO coated on the graphite surface has more capacitive properties. The formation of exfoliated structures as a result of ED-LTO intercalated within PV15 graphite flake and between graphene layers suggests that it is suitable for high-speed energy storage applications. When the charge–discharge behavior of ED-LTO on Pt electrode is examined, it is reversible, although PV15 on Pt electrode shows slightly lower efficiency than graphite (Fig. S5). When the charge–discharge cycles of the electrode are investigated, it is seen that the discharge times are close to each other in multiple cycles and there is serious capacity loss. The hydrogen overvoltage of the Pt electrode is significantly lower compared to the Ti and graphite electrodes. Therefore, the cycle life of the EDT-LTO-coated Pt electrode decreases to 55% after the first 50 cycles and drops to 6% after 1000 charge–discharge cycles (Table 1 and Fig. S5). This significant capacity loss is due to the damage and shedding of the ED-LTO layer caused by the evolution of H_2_ gas during the reduction of residual water in the PC. Consequently, although the Pt electrode is a good inert electrode material for elucidating electrochemical mechanisms, it is not a suitable substrate for battery anode materials naturally. In this study, the Pt electrode was used to better demonstrate the electrodeposition mechanisms of ED-LTO, and these data are described in detail in the first section. When Pt electrode was tested over a wide C rate range such as 13C to 190C, it was found to work efficiently. In summary, when ED-LTO is deposited on Ti and PV15 substrates, the following performances are observed: 105 mA h g^−1^ with 95% capacity retention after 1000 cycles on Ti; 48 mA h g^−1^ with 56% retention on PV15; and 42 mA h g^−1^ with only 6% retention on Pt (Table 1). For comparison, a commercial LTO electrode was prepared and tested (Fig. S6); it delivered a capacity of 88 mA h g^−1^ at 8C. The ED-LTO obtained in our study (105 mA h g^−1^ at 9C) demonstrates a competitive performance compared to the commercial material.

**Table 1:** Capacity, C-rate, and cycle stability of ED-LTO deposited on different substrates

Substrate	Capacity (mA h g^−1^)	C-rate	Cyclic stability after 1000 cycles
Ti	105	9	95
Graphite (PV15)	48	11	56
Pt	42	13	6

EIS analysis of the ED-LTO coated electrode on Pt, Ti, and PV15 graphite surface at various potentials was performed and Nyquist curves (Fig. S7) were presented. The Nyquist diagrams showed a characteristic flattened semicircle in the high frequency region corresponding to the charge transfer resistance (*R*_CT_) and a sloping line in the low frequency region corresponding to the Warburg impedance associated with solid state lithium diffusion. The diameter of the semicircle, *R*_CT_, strongly depended on the electrode potential and showed marked variations, especially around 1.7 V (charged state), the characteristic plateau voltage at which the two-phase transition between the different LTO species occurs. This potential-dependent behavior provides insight into the electrochemical reactions and interfacial processes that determine the performance of ED-LTO as anode material in lithium-ion batteries. The impedance spectra revealed voltage-dependent features throughout the lithiation/delithiation process. EIS parameters were determined by fitting experimental impedance data using the ZSimpWin (version 3.50) impedance program with a suitable electrical equivalent circuit model representing the electrode process (Table 2). The circuits were determined corresponding to the smallest chi-square (*χ*^2^). For Pt and Ti substrates, the *R*_s_(*Q*_SEI_*R*_SEI_)(*Q*_D_(*R*_CT_*W*)) circuit was used, while for PV15, the *R*_s_(*Q*_SEI_*R*_SEI_)(*Q*_D_(*R*_CT_*W*))*C*_DL_ circuit was employed. Here, *R*_s_, *R*_CT_, and *R*_SEI_ represent the solution resistance, the charge transfer resistance, and the resistance to lithium-ion transport within the solid electrolyte interphase (SEI) layer, respectively. A constant phase element (*Q*) was used to account for the non-ideal capacitive behavior of the electric double layer. The Warburg impedance (*W*) describes the system's resistance to the semi-infinite diffusion of Li^+^ ions within the electrode, emphasizing the influence of transport limitations on the electrochemical process. For Pt and Ti electrodes, the resistance values varied depending on the applied potential. At low potentials (1.4 V), the EIS response exhibited pronounced semicircular features in the high frequency region corresponding to charge transfer processes at the electrode/electrolyte interface. The reason why *R*_SEI_ is highest at 1.4 V can be attributed to the formation of an SEI layer on the surface. As the potential increases, *R*_SEI_ gradually decreases because the SEI layer becomes more permeable, and thus the resistance due to SEI becomes negligible. As the potential increased to intermediate values (1.7–2.2 V), a significant decrease in charge transfer resistance was observed, indicating improved kinetics during the lithium insertion/extraction process. The lowest *R*_CT_ observed at 1.7 V can be attributed to the high mobility of Li^+^ diffusion. The decrease in the Warburg impedance (*W*) with increasing potential likewise indicates that Li^+^ diffusion becomes easier. Beyond 1.7 V, the rise in *R*_CT_ suggests that the active phases are approaching lithium saturation, making further Li^+^ insertion more difficult and slowing the charge–transfer reactions. For PV15 (ED-LTO deposited on graphite), the fact that *R*_CT_ and *R*_SEI_ are nearly equal implies the formation of a stable SEI layer, a homogeneous distribution of LTO within the graphite structure, and the absence of any significant side reactions in the 1.7–3.2 V window other than graphite intercalation. When Pt, Ti, and PV15 are compared, the highest capacitance (*Q*_D_) value is obtained on the Ti electrode. This can be explained by the Ti substrate's ability to promote more ordered ED-LTO growth, provide a more stable interfacial layer, and thereby ensure stronger adhesion of the phases to the surface.

**Table 2:** EIS parameters obtained from data given in Fig. S7 (from charged state to discharged state: 1.4 V, 1.7 V, 2.7 V, 2.9 V, 3.2 V respectively) using the *R*_s_(*Q*_1_*R*_SEI_)(*Q*_2_(*R*_CT_*W*)) and *R*_s_(*Q*_SEI_*R*_SEI_)(*Q*_D_(*R*_CT_*W*))*C*_DL_ circuits

Pt	*R*_s_ (mΩ cm^2^)	*Q*_SEI_ (mF s n^−1^ cm^−2^)	*R*_SEI_ (Ω cm^2^)	*Q*_D_ (mF s n^−1^ cm^−2^)	*R*_CT_ (Ω cm^2^)	*W* (mΩ s^−1/2^ cm^−2^)	*C* (mF cm^−2^)	*χ* ^2^ × (10^−3^)
1.4 V	32.8	5384.4	210.6	0.3	101.4	26.9		3.0
1.7 V	27.3	166.7	13.3	0.6	17.9	25.6		4.5
2.2 V	25.0	19.2	0.1	0.7	187.2	13.5		3.2
2.7 V	17.9	13.7	0.2	0.9	202.8	4.6		2.3
								
**Ti**								0.0
1.4 V	375.9	676.8	51.8	0.1	145.0	105.8		3.3
1.7 V	354.6	2608.5	5.3	5.9	46.8	25.0		5.4
2.2 V	290.8	3.7	0.4	1.0	70.2	8.6		2.9
2.7 V	198.6	2.4	0.4	0.9	106.4	5.1		3.0
								
**PV15**								0.0
1.7 V	4700	0.5	10.6	0.5	27.0	21.0	92.0	0.5
2.0 V	4700	0.5	10.6	0.5	26.0	19.0	73.0	0.6
2.3 V	4300	0.6	11.0	0.4	25.0	17.0	57.0	0.6
2.6 V	4400	0.6	11.0	0.5	24.0	15.0	45.0	0.6
2.9 V	4500	0.6	11.0	0.7	25.0	15.0	45.0	0.6
3.2 V	4300	0.7	12.0	0.6	25.0	17.0	55.0	0.5

### Spectroscopic characterization

**Fig. 7:**
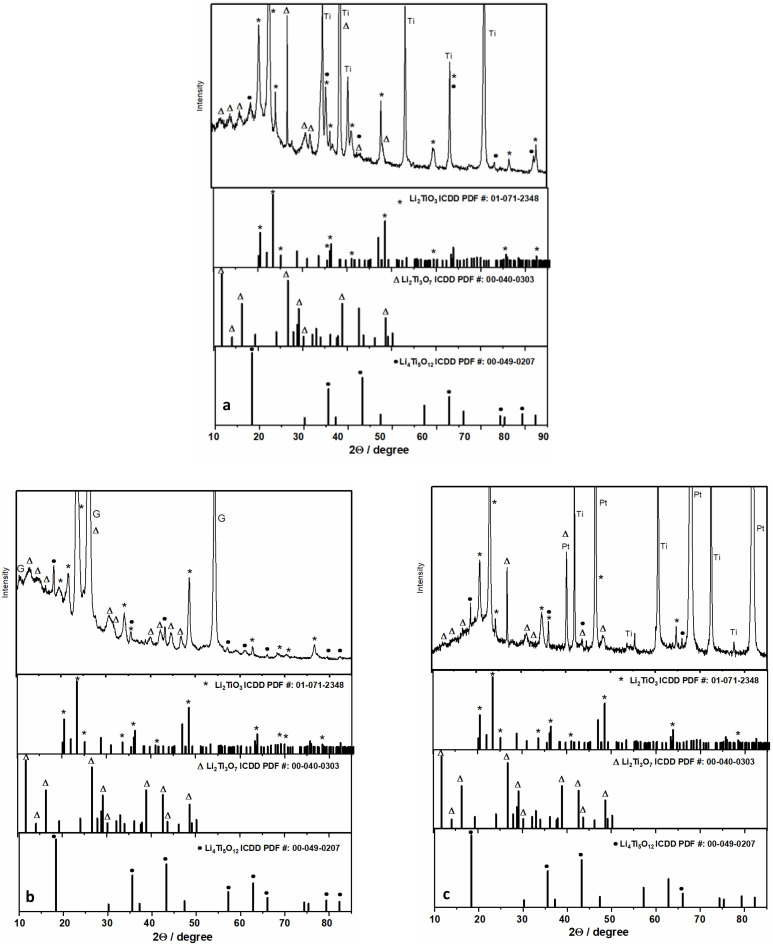
XRD pattern of ED-LTO coated on (a) Ti, (b) PV15, (c) and Pt substrates.

XRD analysis of the LTO coated Ti, PV15 graphite composite (G), and Pt was conducted (Fig. 7), the observed peaks arose from Li_2_TiO_3_ (ICDD PDF #: 01-071-2348), Li_2_Ti_3_O_7_ (ICDD PDF #: 00-040-0303), and Li_4_Ti_5_O_12_ (ICDD PDF #: 00-049-0207) in addition to the substrate peak of Ti, Pt and G. In the case of using a platinum substrate, metallic Ti was obtained besides LTO phases. Similarly, the deposition of Ti on the Au and Cu electrode surface in PC solution at constant current has been reported in the literature.^24^ To compare ED-LTO, the commercial LTO sample was analyzed using XRD (Fig. S8) and only showed the presence of Li_4_Ti_5_O_12_ (ICDD PDF #: 00-049-0207). To monitor the cycling stability of the ED-LTO coatings after charge–discharge, an XRD analysis of the ED-LTO-coated Ti electrode was recorded after 1000 cycles at 38C (Fig. S9). The disappearance of the peaks at low angles suggests partial amorphization of the surface; however, the absence of significant changes in the remaining peaks shows that the bulk crystal structure was preserved without degradation. This result indicates that ED-LTO maintains its structural stability even at high current rates.

XPS was used to characterize the chemical state of elements in the LTO samples (Fig. S10), the presence of Li 1s, Ti 2p, and O 1s confirms the formation of LTO. The Ti 2p spectrum shows four peaks at binding energies of 464.0 eV and 458.3 eV, 461.0 eV and 456.1 eV, corresponding to Ti 2p_3/2_ and 2p_1/2_ of Ti^4+^, Ti 2p_3/2_ and 2p_1/2_ of Ti^3+^, respectively O 1s was deconvoluted, resulting in two peak values at 531. eV and 533.1 eV, corresponding to lattice O, and the formation of oxygen vacancies, respectively. The presence of oxygen vacancies enhances the conductivity and kinetics of LTO, thereby yielding a material with better electrochemical activity.^30,31^

**Fig. 8:**
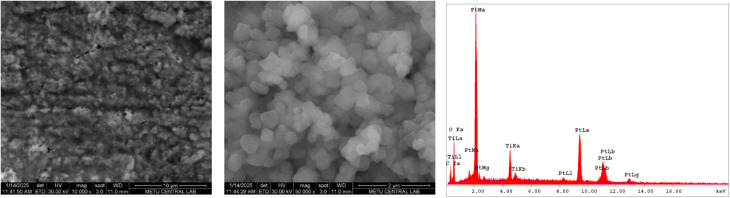
FESEM-SE images and EDX spectrum of LTO-coated Pt.

The morphology of ED-LTO deposited on Pt, Ti and PV15 was investigated using FESEM. When the commercial LTO sample was also analyzed, 100–500 nm sized spherical particles were observed (Fig. S8). Fig. 8 shows FESEM-SE images of LTO deposited on Pt, revealing a network of agglomerated nanospheres with characteristic spinel morphology.^31^ The nanoparticles form interconnected clusters with uniform size distribution. EDX spectra of the coating exhibited Ti and O, alongside Pt from the substrate while Li was not observed due to inherently low characteristic X-ray energy.^31,32^ This nanoscale morphology enhances lithium-ion diffusion kinetics, crucial for high-performance lithium-ion battery applications. For the Ti substrate, FESEM displays a porous network structure composed of agglomerated LTO particles with irregular spherical morphology (Fig. S11). The morphology facilitated enhanced electrolyte penetration and lithium-ion transport during charge–discharge cycles. The EDX analysis confirms the presence of Ti and O, consistent with the formation of lithium titanate phases. These nanospheres exhibit interconnected structures with void spaces between particle clusters, which makes lithium-ion transport easier.^33^ Pt substrates favor spinel nucleation, while Ti promotes ramsdellite growth. For LTO deposited on PV15 graphite composite, irregular cluster-like structures with interconnected nanospheres were obtained in addition to the partial penetration of LTO species into the graphite layers (Fig. S12). This LTO intercalation causes the delamination or exfoliation of the surface graphite layers, which may facilitate rapid lithium-ion diffusion and electrolyte access. After 1000 cycles at high C-rates, the surfaces of the ED-LTO-coated Ti and PV15 electrodes were examined by FESEM (Fig. S13 and S14). The SEM images reveal that the nanostructured morphology is largely preserved; however, partial agglomeration and increased surface roughness due to the presence of nanorods are observed in some regions, which can be attributed to rapid Li^+^ diffusion and volumetric changes. Such morphological changes, as also reported in the literature,^34,35^ can be associated with fast lithium transport and repeated volume changes.

LTO-coated Pt and LTO-coated Ti were analyzed using RAMAN (Fig. S15) to compare with commercial LTO powder, TiO_2_ powder (rutile), and bare Ti metal. For LTO-coated Pt and LTO-coated Ti samples, the Raman peaks at ∼266 cm^−1^ and ∼695 cm^−1^ are assigned to Ti–O bending and stretching vibrations in TiO_6_ octahedral, while the peak at ∼440 cm^−1^ is assigned to Li–O stretching vibrations in LiO_4_ tetrahedral of Li_4_Ti_5_O_12_ (LTO), respectively.^36–40^ The results confirm the formation of ED-LTO.

The full cell was assembled using LiFePO_4_ and ED-LTO, and the resulting cell was tested at various C-rates (Fig. S16). Full cell voltage was measured as 1.85 V, a specific capacity of 85 mA h g^−1^ was achieved at 8C. These values are consistent with those reported in the literature for LTO synthesized *via* thermal methods (69–78 Ah g^−1^ (ref. 41–43)). To investigate interface stability and SEI layer formation, XPS depth profile measurements were performed on ED-LTO electrodes in the charged state after a long charge–discharge cycle (Fig. S17). Despite the etching process, no significant change in the intensity of Li, Ti, and O peaks was observed on the surface and in the lower layers, indicating that the spinel crystal structure of LTO was preserved and maintained its stability. This situation shows that lithium is retained not only on the surface but also within the electrode volume and does not easily detach from the structure. The 95% capacity retention achieved after 1000 cycles is directly related to this stable SEI layer. While a weakening of the Li signal on the surface is expected after discharge under normal conditions, the persistence of the Li signal throughout the entire depth profile suggests that the Li_4_Ti_5_O_12_ → Li_7_Ti_5_O_12_ transformation is not completely reversible and that a certain amount of Li^+^ ions are trapped within the structure.

## Conclusions

This study successfully demonstrated a novel room-temperature electrodeposition method for synthesizing lithium titanate (LTO) anode materials directly on various electrode surfaces using titanyl compounds in propylene carbonate solutions. This approach represents a significant advancement over conventional high-temperature (600–1100 °C) synthesis methods typically employed for LTO production. The electrochemical mechanism of LTO formation was elucidated through cyclic voltammetry studies, revealing that TiO^2+^ ions undergo reduction to unstable TiO^+^ species at approximately 2.9 V (*vs.* Li/Li^+^). These species subsequently react with hydroxide ions generated from water electroreduction to form TiO(OH)_2_ and TiOOH intermediates, interacting with lithium ions to produce various LTO phases. The simultaneous electroreduction between 2.4 V and 1.2 V facilitates lithium insertion during deposition, resulting in a composite structure containing Li_4_Ti_5_O_12_, Li_2_Ti_3_O_7_, and Li_2_Ti_3_O_7_/Li_2_TiO_3_. Comprehensive characterization using XRD, XPS, Raman spectroscopy, and SEM-EDX confirmed the formation of multiple LTO phases with distinct morphologies dependent on the substrate material. The XPS analysis revealed the presence of both Ti^4+^ and Ti^3+^ oxidation states and oxygen vacancies, enhancing the electrical conductivity of the coating. SEM imaging showed that electrodeposited LTO on Pt substrates formed interconnected nanosphere networks with characteristic spinel morphology, while Ti substrates yielded porous structures, and graphite substrates exhibited partial intercalation of LTO between graphene layers. Electrochemical performance testing demonstrated that the electrodeposited LTO exhibits excellent functional properties as an anode material. Charge–discharge profiles showed characteristic plateaus around 1.7 V, with capacities reaching approximately 166 mA h g^−1^ at a 2C rate. Remarkably, the ED-LTO electrodes maintained noticeable performance across a wide range of C rates, from 2C to as high as 72C on Ti substrates and up to 230C on graphite substrates. Long-term cycling tests revealed outstanding stability with 95% capacity retention after 1000 cycles at high C-rates, indicating exceptional structural integrity during lithium insertion/extraction. Depth-profile XPS conducted after prolonged cycling showed unchanging Li, Ti, and O signals throughout the film thickness, demonstrating a stable SEI layer and the preservation of the spinel framework that supports the high-capacity retention. The substrate material significantly influenced the morphology, phase composition, and electrochemical behavior of the deposited LTO. Platinum substrates favored spinel phase formation, whereas titanium promoted ramsdellite growth. The partial intercalation of LTO into graphite layers created exfoliated structures that exhibited enhanced capacitive properties suitable for high-rate applications. When full cell constructed with a LiFePO_4_ cathode, the ED-LTO anode delivered an operating voltage of 1.85 V and a reversible capacity of 85 mA h g^−1^ at 8C, indicating its practical applicability in high-power lithium-ion batteries. The direct electrodeposition method developed in this study offers several advantages over conventional synthesis approaches, including room temperature processing, single-step fabrication, elimination of high-temperature calcination, and the ability to control morphology through electrochemical parameters. These features make the process more energy-efficient and potentially scalable for industrial applications. This work opens new avenues for the development of lithium-ion battery materials through electrochemical routes and demonstrates that high-performance LTO anodes can be produced through direct electrodeposition. Future research could optimize the electrodeposition parameters to enhance specific capacity, investigate the effect of various additives on the LTO structure, and scale up the process for practical battery applications.

## Author contributions

Fatma Çambay Kuban contributed to the conceptualization, methodology, data collection, analysis, writing-original draft preparation, and writing-review and editing. Kadir Pekmez contributed to supervision, methodology, writing-review and editing, and critical revisions of the manuscript. The manuscript is a part of the PhD thesis of Fatma Çambay Kuban.

## Conflicts of interest

There are no conflicts to declare.

## Supplementary Material

RA-015-D5RA06413A-s001

## Data Availability

All data supporting the findings of this study are available within the article and its SI files. Raw output files and additional data generated during this study are available from the corresponding author upon reasonable request. Supplementary information is available. See DOI: https://doi.org/10.1039/d5ra06413a.
