# Supplementary material for: Direct electrodeposition of lithium titanate as a lithium-ion battery anode active material in propylene carbonate solution containing titanyl compounds
Source: RSC Adv. 2025 Sep 25;15(42):35356–67. doi: 10.1039/d5ra06413a (PMC12461247; doi:10.1039/d5ra06413a)
Supplement: RA-015-D5RA06413A-s001 [file RA-015-D5RA06413A-s001.pdf]

Supplementary Information (SI) for RSC Advances.
This journal is © The Royal Society of Chemistry 2025

Supplementary Materials

Direct Electrodeposition of Lithium Titanate as a Lithium-ion Battery Anode Active Material in Propylene Carbonate Solution Containing Titanyl compounds

Fatma Çambay Kuban[a] and Kadir Pekmez *[b]

[a]*Graduate School of Science and Engineering, Nanotechnology and Nanomedicine Program, Hacettepe University, Ankara, Turkey*
[b]*Department of Chemistry, Hacettepe University, Ankara, Turkey*
*Corresponding Author E-mail Address: pekmez@hacettepe.edu.tr

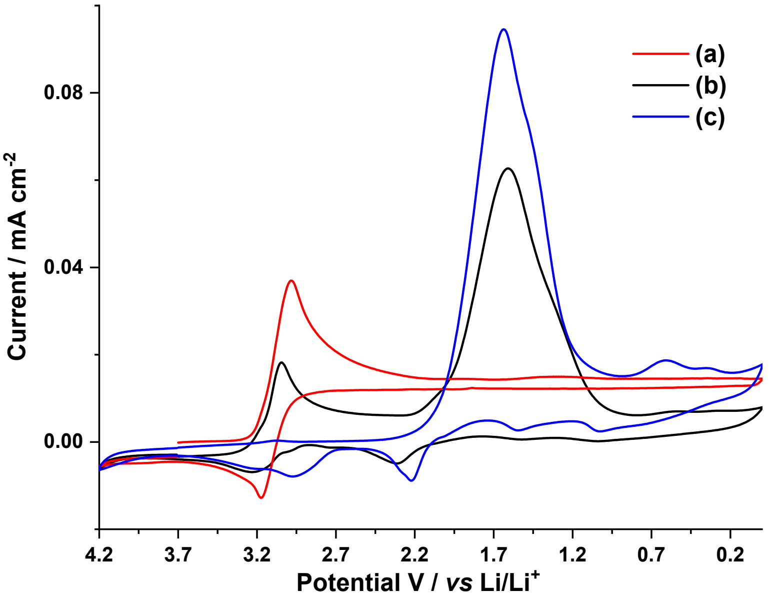

Figure S1. (a). CV obtained on Pt disc electrode in 0.100 M $TiO(ClO_4)_2$ PC solution, **(b)** after addition of 0.1 M water and 0.5 M $LiClO_4$ to the solution in a. **c)** CV of solutions obtained by adding solid LiOH to the b solution, *v*: 100 mV/s.

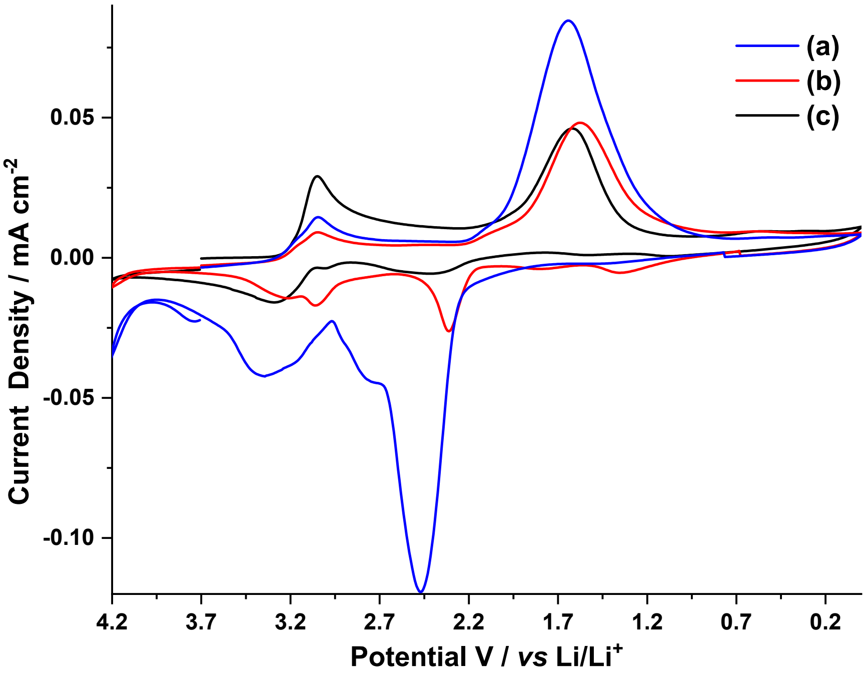

Figure S2. (a) CV obtained at Pt disc electrode in PC solution containing 0.10 M $TiO(ClO_4)_2$, 0.10 M water and 0.5 M $LiClO_4$ **.** At 0.7 V, the scan was stopped and electrolyzed at this voltage for **(b)** 2 min and **(c)** 10 min and then the scan was completed.

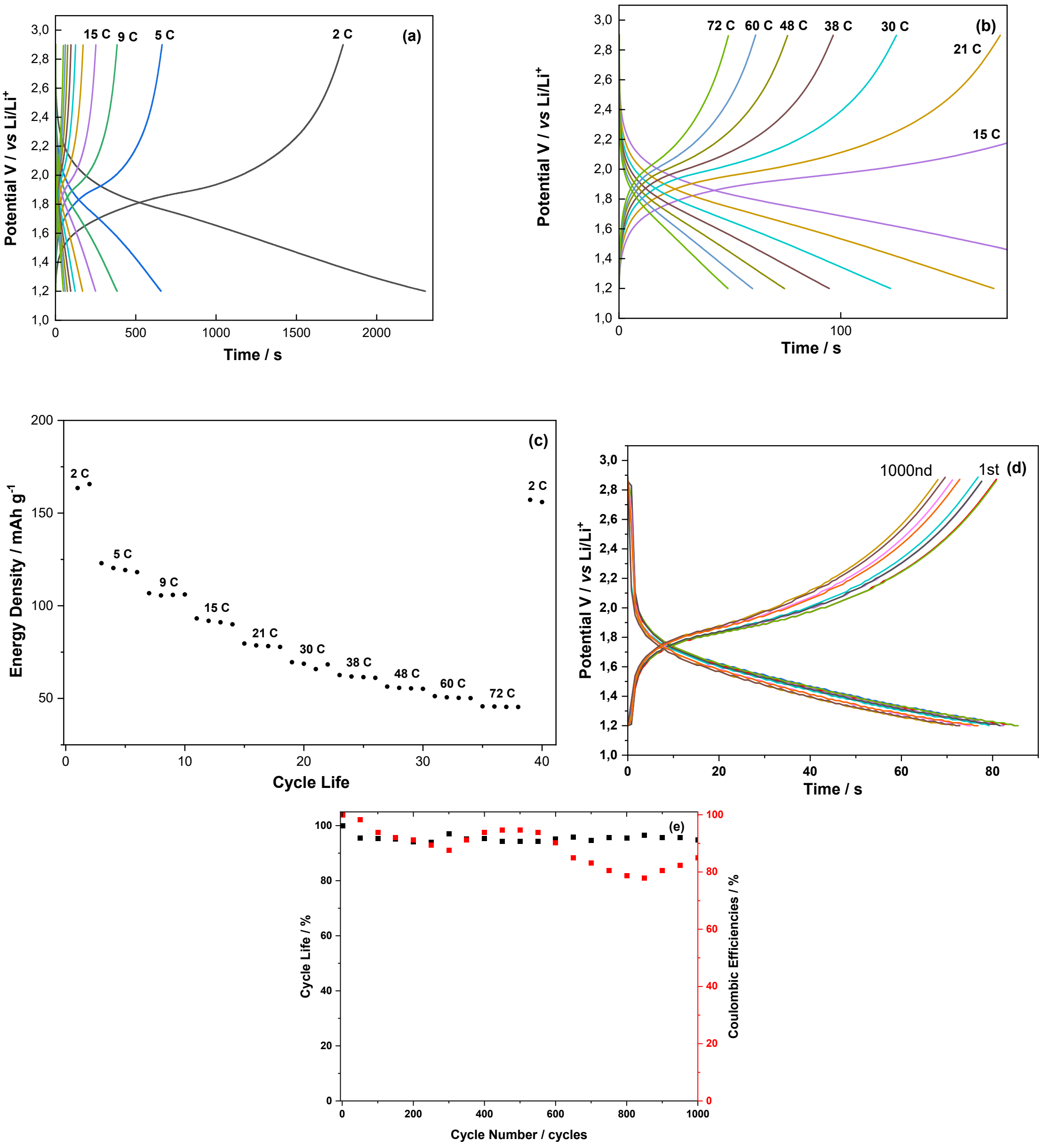

Figure S3. Charge-discharge curves recorded in 1.0 M $LiClO_4$-PC blank solution **(a)** at 2 C - 72 C rates, **(b)** rate capability data (2 C – 72 C), **(c)** charge-discharge curves after every 100 cycles during 1000 cycles, and **(d)** cycle life and coulombic efficiencies at 38 C during 1000 cycles of ED-LTO obtained by coating on Ti sheet electrode surface for 30 min by potential-limited galvanostatic electrolysis in PC solution containing 0.10 M $TiO(ClO_4)_2$, 0.50 M $LiClO_4$, and 0.10 M water.

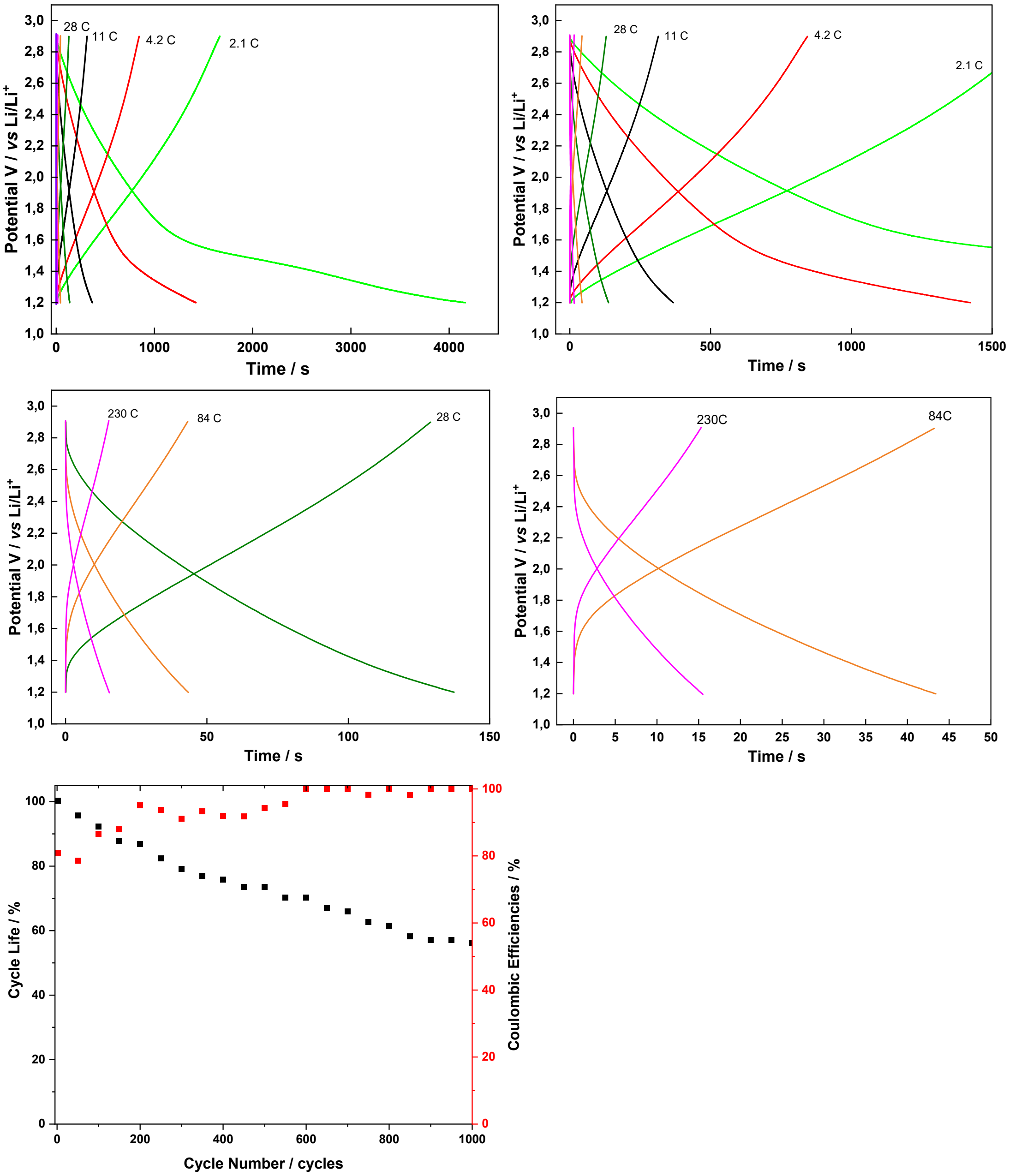

Figure S4. Charge-discharge curves recorded 1.0 M $LiClO_4$-PC blank solution at various current densities (C-rate values 2.1 C – 230 C) of ED-LTO obtained by coating on PV15 graphite composite electrode surface for 45 min by potential-limited galvanostatic electrolysis in PC solution containing 0.10 M $TiO(ClO_4)_2$, 0.50 M $LiClO_4$, and 0.10 M water.
The cycle life and coulombic efficiencies at 40 C during 1000 cycles of ED-LTO obtained by coating on PV15 sheet electrode surface

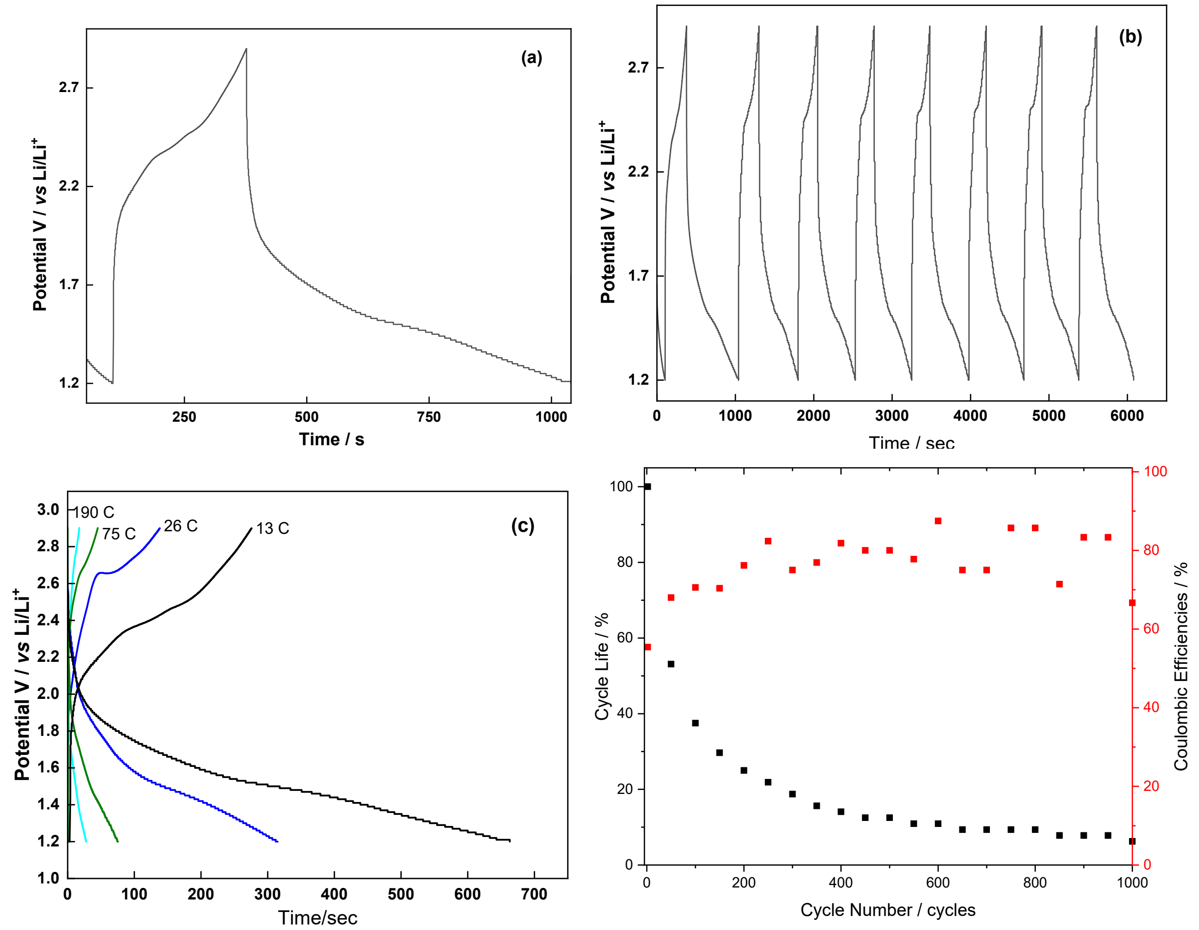

Figure S5. a) Single cycle and **b)** multi-cycle charge-discharge curves, and c) charge-discharge curves recorded in 1.0 M $LiClO_4$-PC blank solution at various current densities (C-rate values 13 C – 190 C) of ED-LTO obtained by coating on Pt sheet electrode surface for 30 min by potential-limited galvanostatic electrolysis in PC solution containing 0.10 M $TiO(ClO_4)_2$, 0.50 M $LiClO_4$, and 0.10 M water.
The cycle life and coulombic efficiencies at 40 C during 1000 cycles of ED-LTO obtained by coating on Pt electrode surface

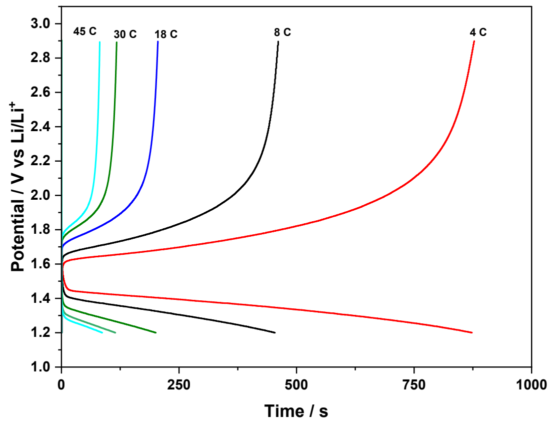

Figure S6. Charge-discharge curves recorded in 0.1 M $LiClO_4$-PC blank solution at various current densities (C-rate values 4 C – 45 C) of commercial LTO ($Li_4Ti_5O_{12}$).

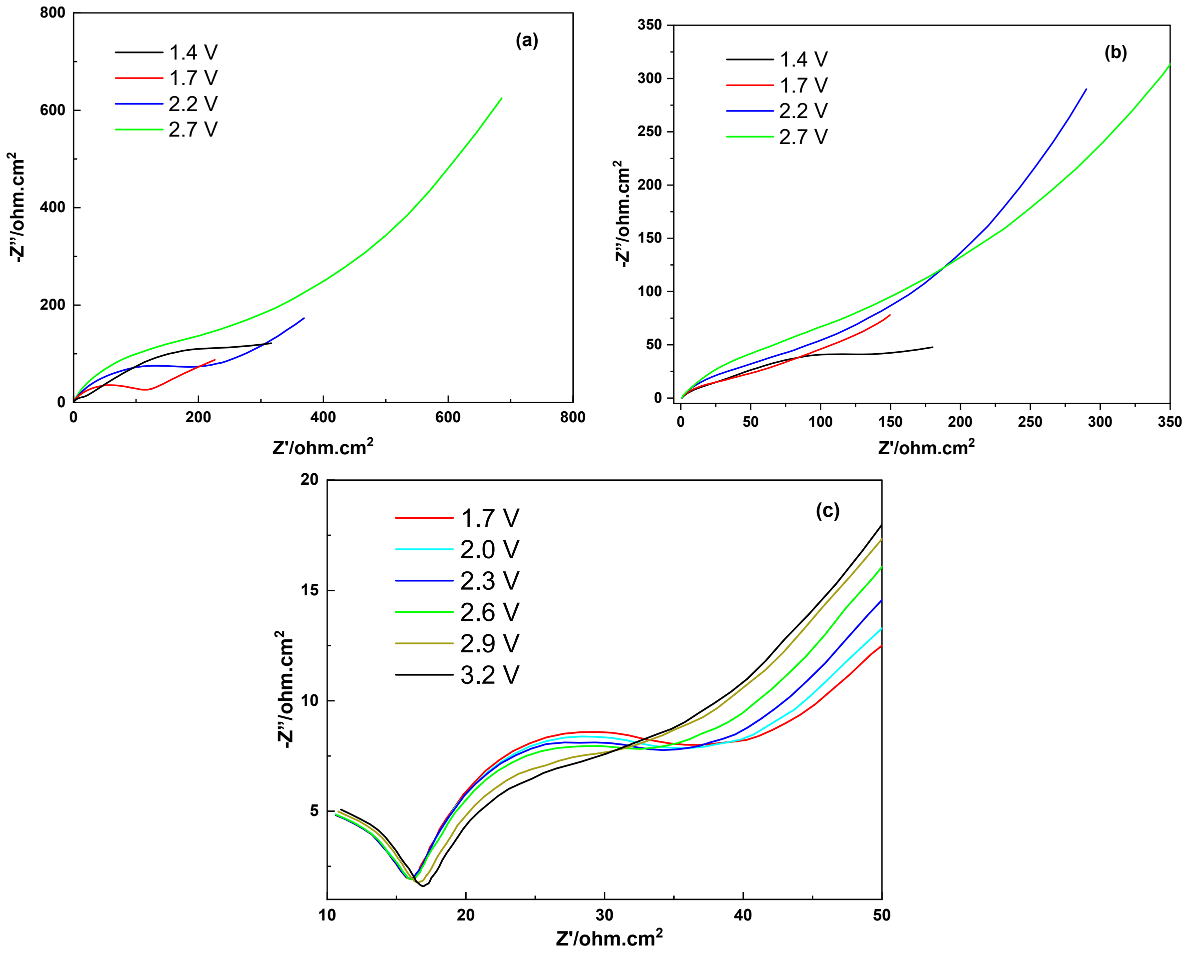

Figure S7. Nyquist curves recorded at various potentials (from charged state to discharged state: 1.4V, 1.7V, 2.7V, 2.9V, 3.2V respectively) of ED-LTO obtained by coating on a) Pt disc and b) Ti disc electrode surface for 30 min, c) PV15 electrode surface for 45 min by potential-limited galvanostatic electrolysis in PC solution containing 0.10 M $TiO(ClO_4)_2$, 0.50 M $LiClO_4$, and 0.10 M water.

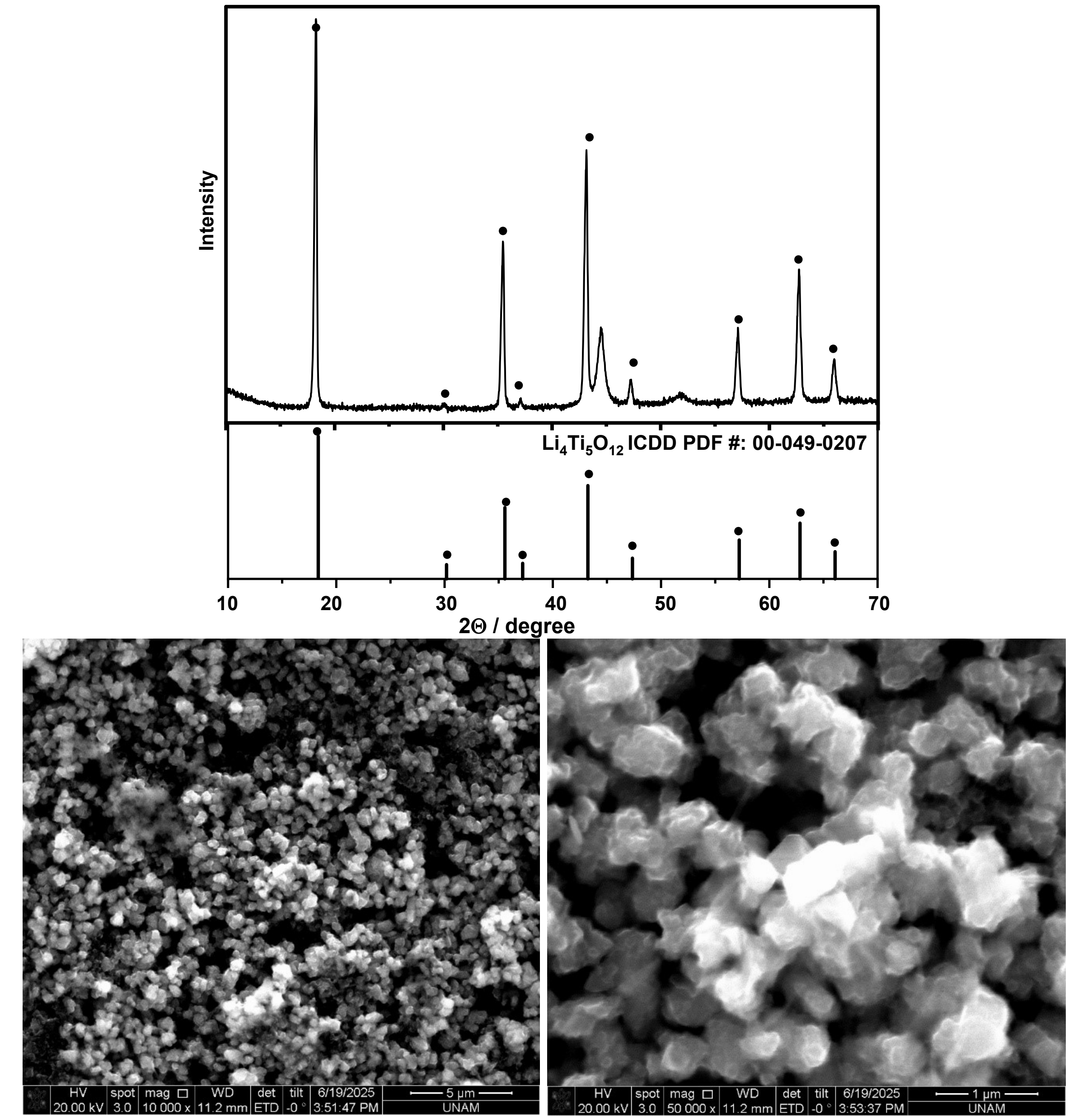

Figure S8. XRD pattern and FESEM-SE images of commercial LTO ($Li_4Ti_5O_{12}$).

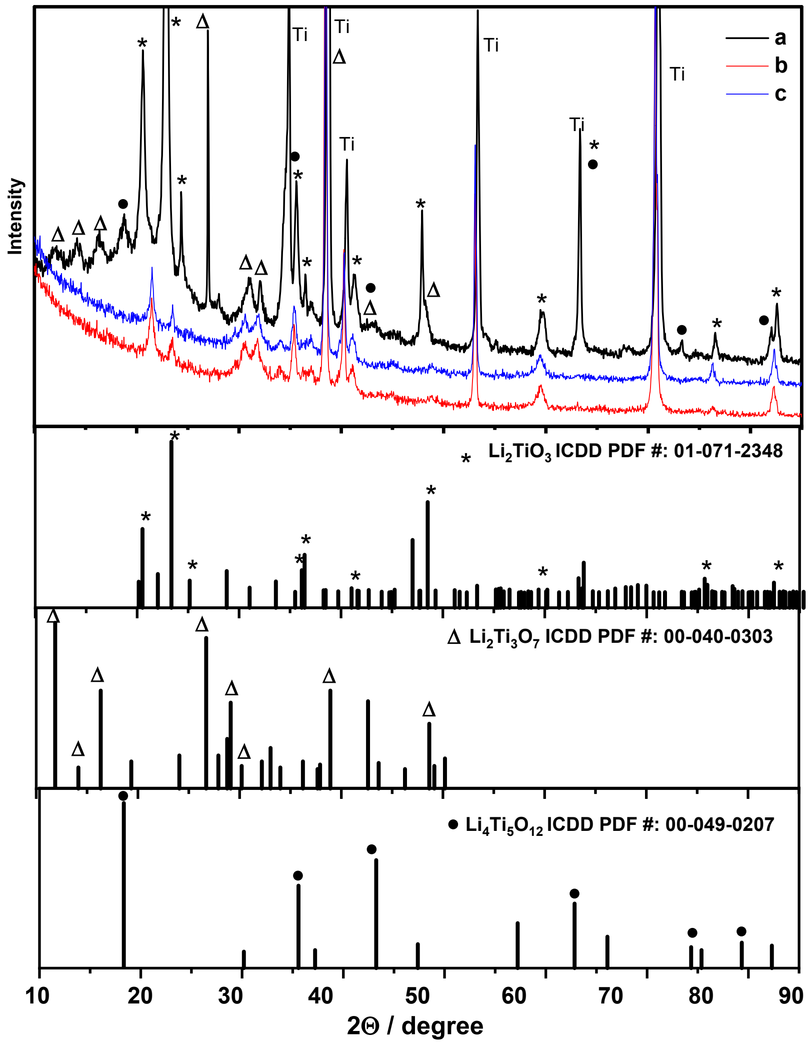

Figure S9. XRD pattern of ED-LTO coated Ti **(a)** before charge-discharge tests, after 1000 cycle charge-discharge at 38 C in **(b)** charged state and **(c)** discharged state.

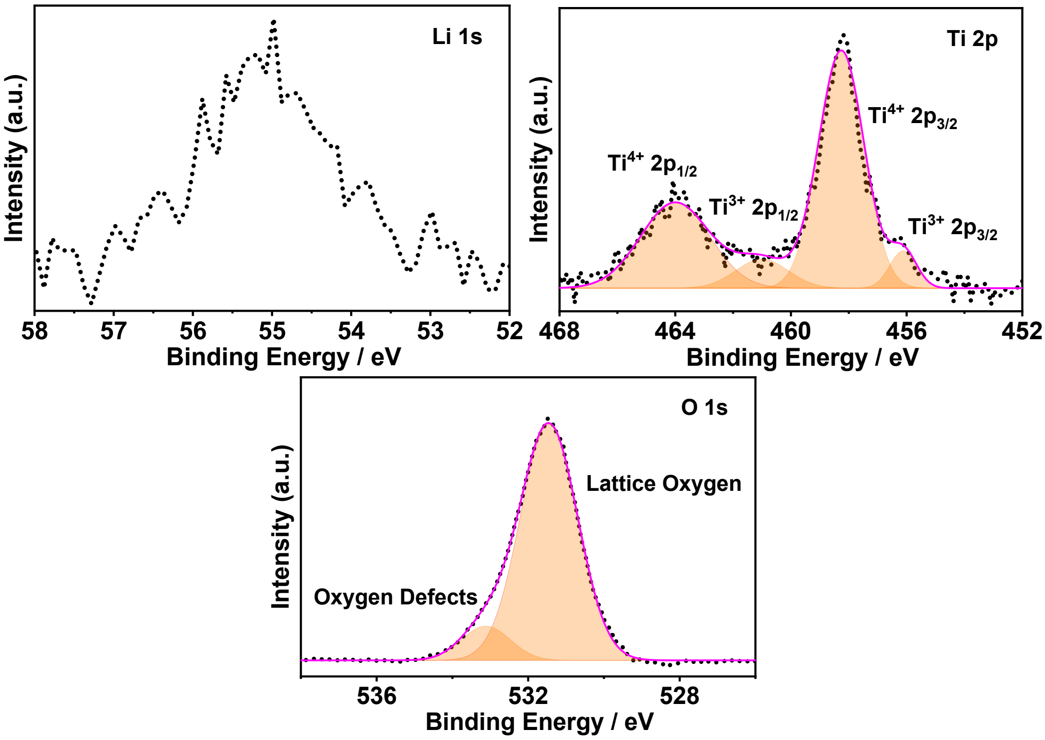

Figure S10. Li1s, Ti 2p, and O 1s of XPS spectra for ED-LTO-coated Pt.

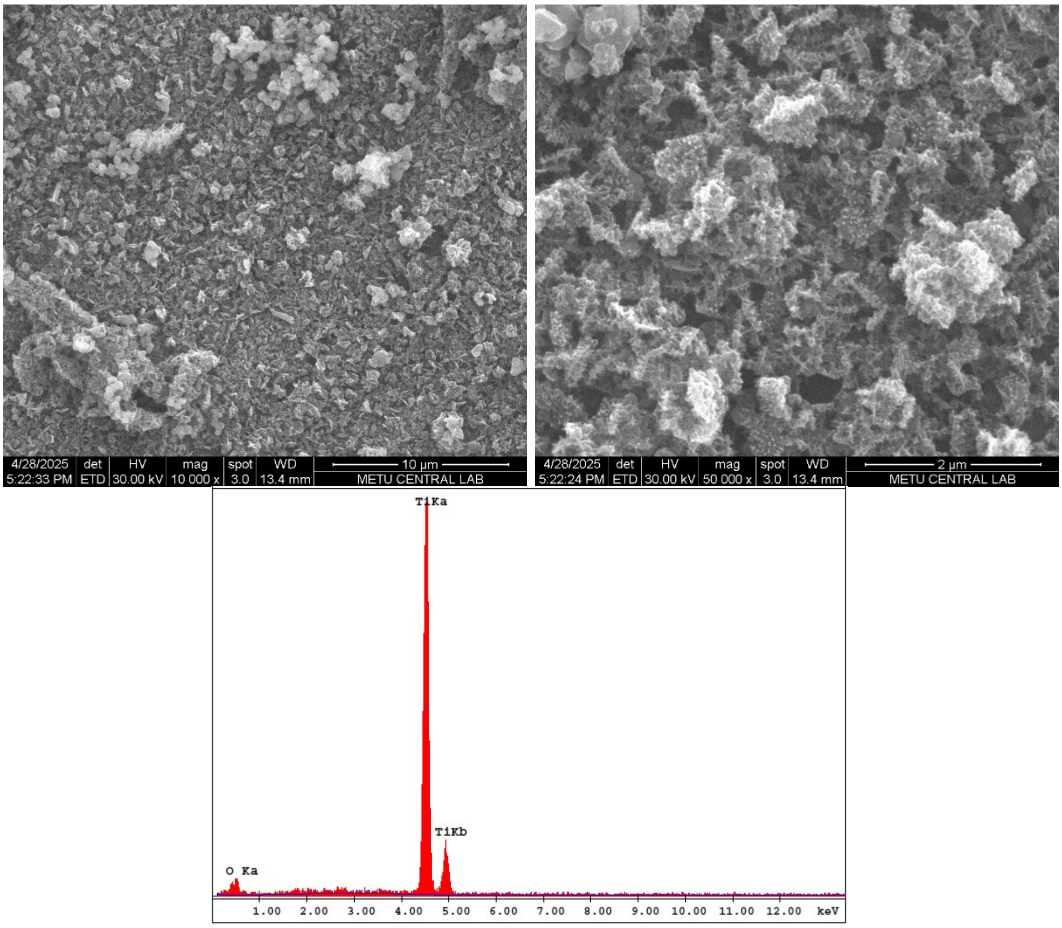

Figure S11. FESEM-SE images and EDX spectrum of ED-LTO-coated Ti.

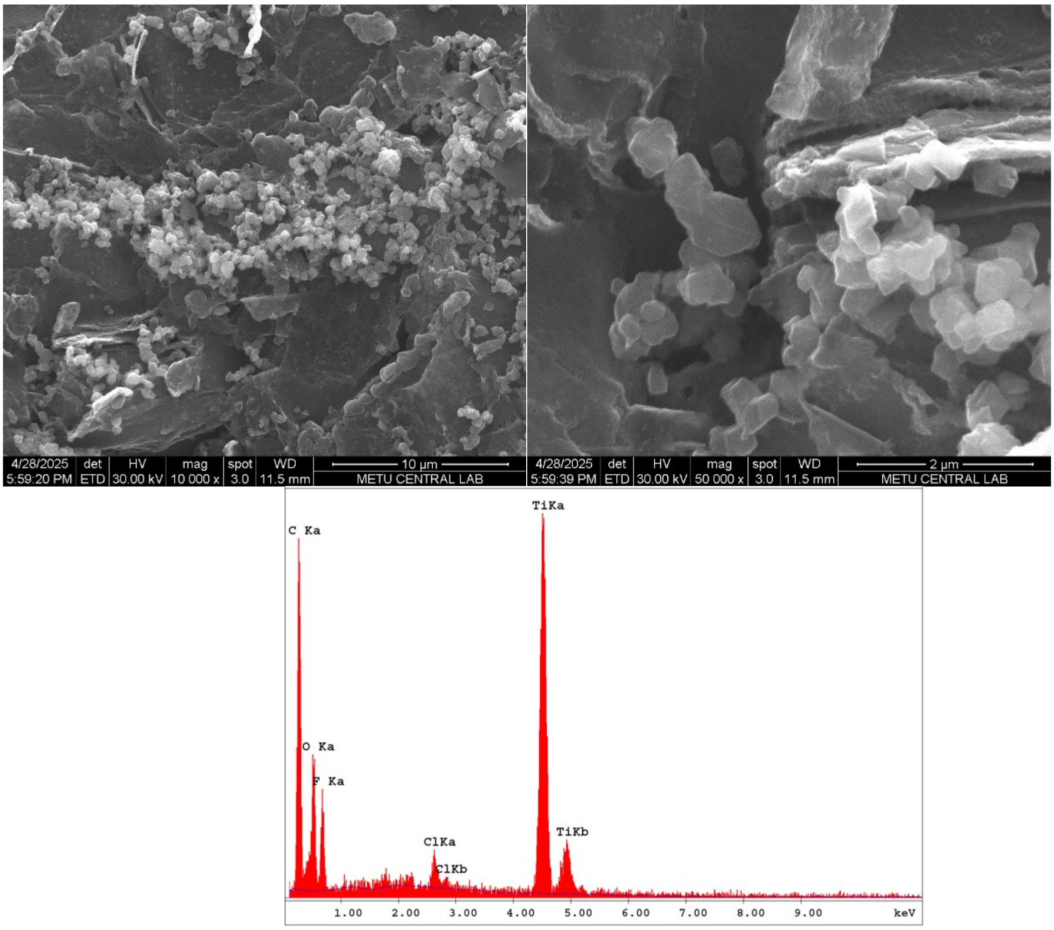

Figure S12. FESEM-SE images and EDX spectrum of ED-LTO-coated PV15.

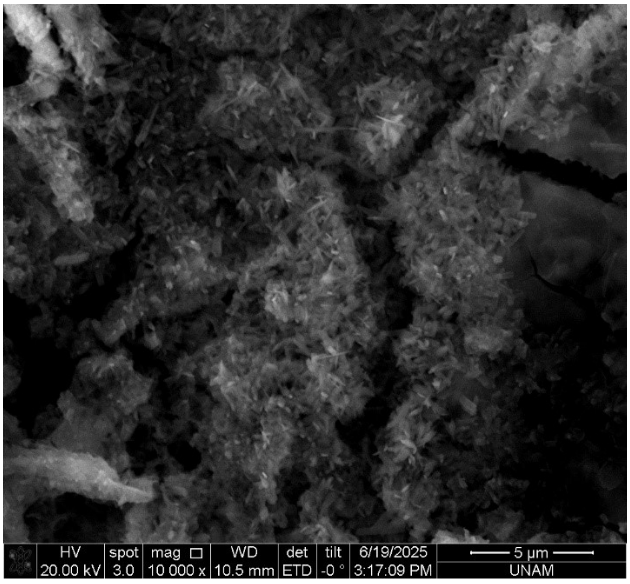

Figure S13. FESEM-SE images and EDX spectrum of ED-LTO-coated Ti after 1000 cycle charge discharge at 38 C.

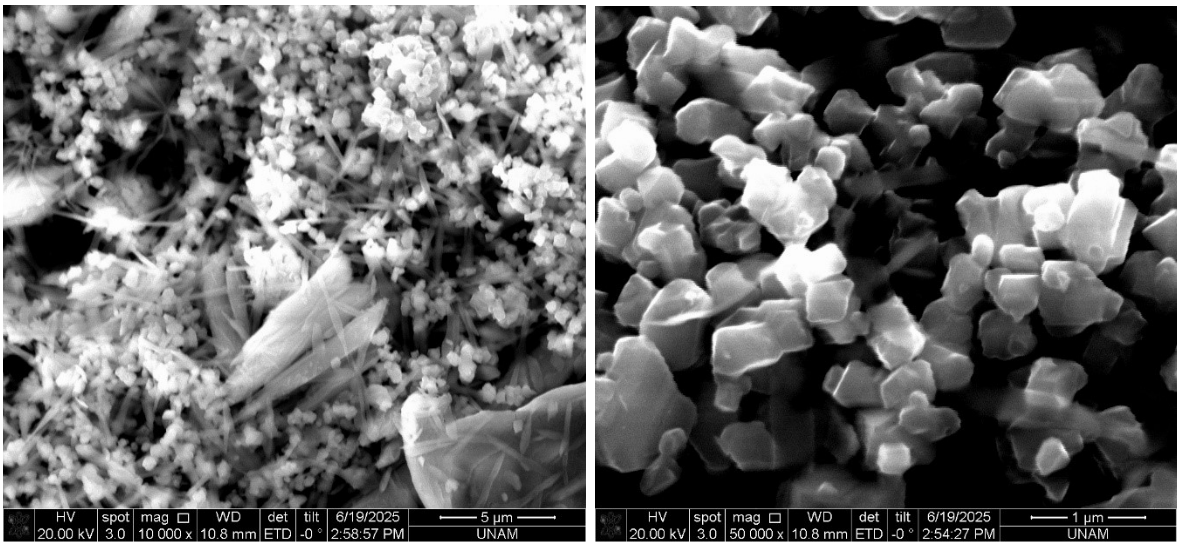

Figure S14. FESEM-SE images and EDX spectrum of ED-LTO-coated PV15 after 1000 cycle charge discharge at 230 C.

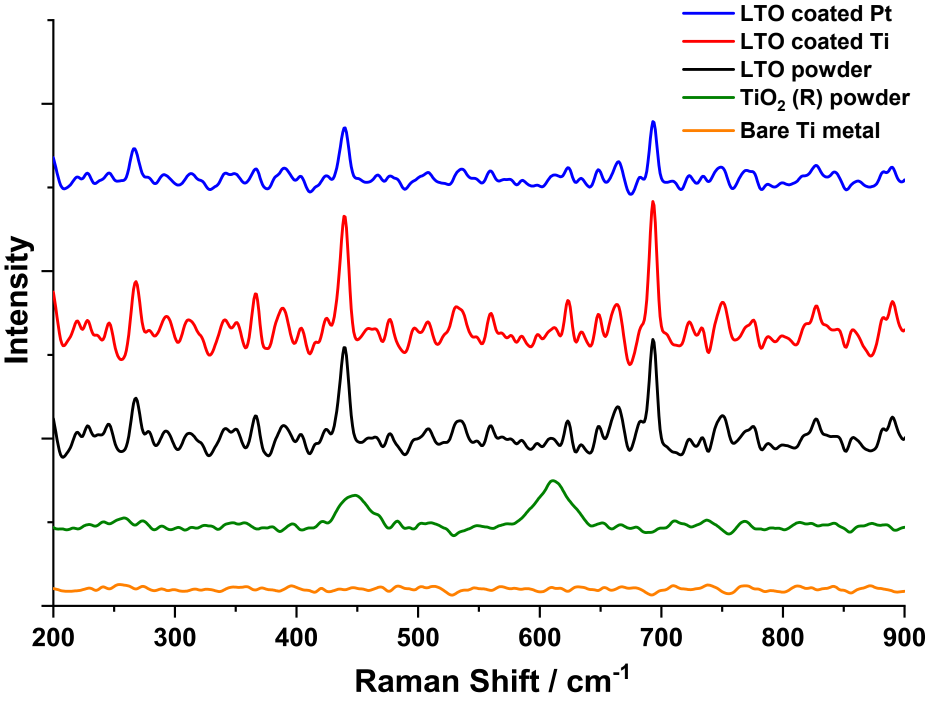

Figure S15. Raman spectra of ED-LTO-coated Pt and ED-LTO-coated Ti, commercial LTO powder, TiO_2 powder, and bare Ti metal.

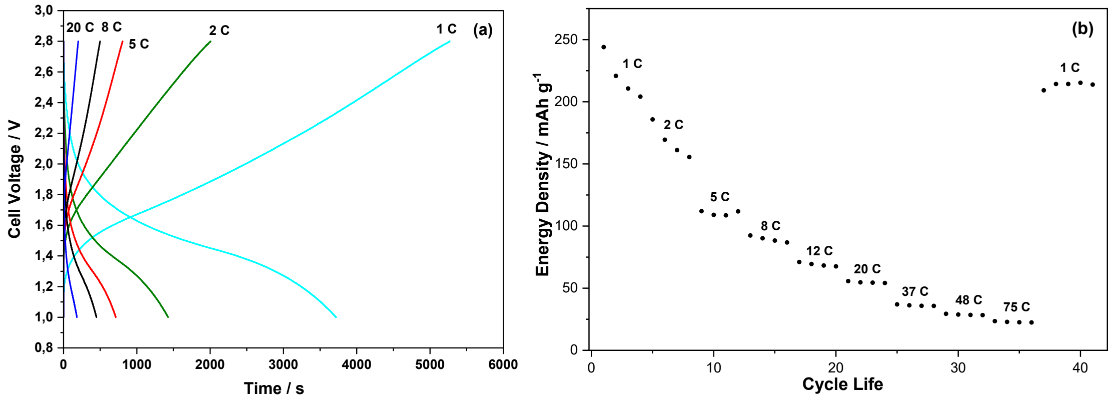

Figure S16. Charge-discharge curves recorded at various current densities (C-rate values) of $LiFePO_4$//ED-LTO full cell in 1.0 M $LiClO_4$-PC blank solution **(a)** at 1 C - 20 C rates **(b)** rate capability data (at 1 C- 75 C rates) recorded in 1.0 M $LiClO_4$-PC blank solution

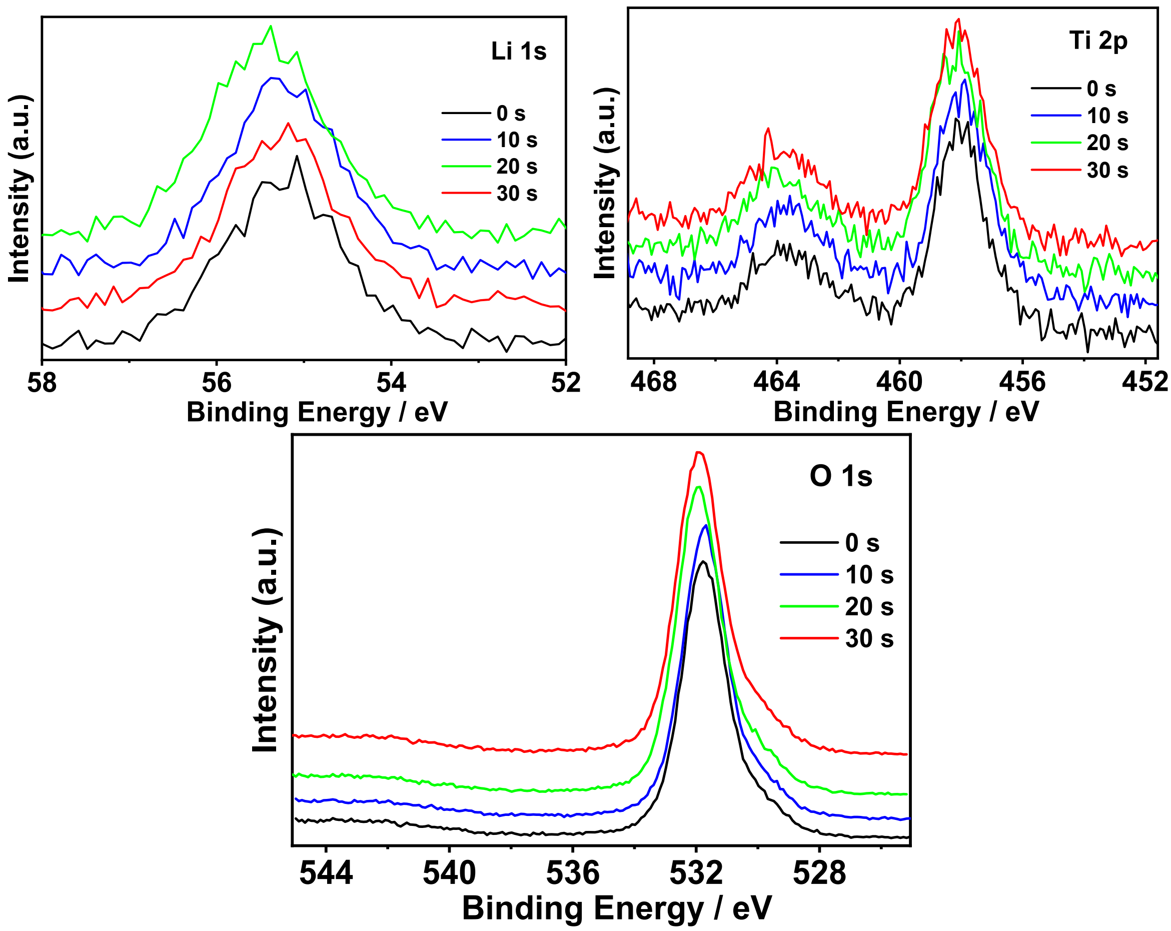

Figure S17. Li1s, Ti 2p, and O 1s values from depth profiling XPS measurements after 10 s, 20 s, and 30 s etching for ED-LTO electrodes in a charged state exposed to a long charge-discharge cycle.
